# Stabilization of SQLE mRNA by WTAP/FTO/IGF2BP3-dependent manner in HGSOC: implications for metabolism, stemness, and progression

**DOI:** 10.1038/s41419-024-07257-6

**Published:** 2024-12-01

**Authors:** Rui Hou, Xinrui Sun, Shiyao Cao, Yadong Wang, Luo Jiang

**Affiliations:** 1 Department of Obstetrics and Gynecology, Shengjing Hospital of China Medical University, Shenyang, China; 2 Department of Ultrasound, Shengjing Hospital of China Medical University, Shenyang, China

**Keywords:** Cell growth, Oncogenes

## Abstract

The metabolic reprogramming in high-grade serous ovarian carcinoma (HGSOC) affects the tumor stemness, which mediates tumor recurrence and progression. Knowledge of the stemness and metabolic characteristics of HGSOC is insufficient. Squalene epoxidase (SQLE), a key enzyme in cholesterol metabolism, was significantly upregulated in HGSOC samples with a fold change of about 4 in the RNA sequencing analysis. SQLE was positively related to peritoneal metastasis and poor prognosis of HGSOC patients. Functionally, SQLE drove cancer cell proliferation and inhibited apoptosis to accelerate HGSOC growth. SQLE was highly expressed in ALDH^+^CD133^+^ FACS-sorted cells derived from HGSOC cells and ovarian cancer stem cells (OCSCs)-enriched tumorspheres. SQLE overexpression resulted in enhanced CSC-like properties, including increased tumorsphere formation and stemness markers expression. In vivo, SQLE not only promoted cell line-derived xenografts growth but extended the OCSCs subpopulation of single-cell suspension. Moreover, non-targeted metabolomics profiling from UPLC-MS/MS system identified 90 differential metabolites responding to SQLE overexpression in HGSOC cells. Among them, the dysfunctional metabolisms of cholesterol and glutathione were involved in the maintenance of HGSOC stemness. Previous studies showed the alteration of N6-Methyladenosine (m6A) modification in HGSOC development. Herein, the m6A modification in the 3’UTR and CDS regions of SQLE mRNA was increased due to upregulated methyltransferases WTAP and downregulated demethylases FTO, which was recognized by m6A-binding proteins IGF2BP3, rather than IGF2BP1 or IGF2BP2, thereby stabilizing the SQLE mRNA. These results suggested that SQLE was a novel potential clinical marker for predicting the HGSOC development and prognosis, as well as a potential therapeutic target of HGSOC.

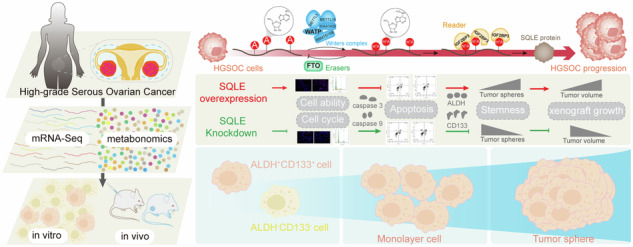

## Introduction

Ovarian carcinomas have the highest mortality rate among gynecological cancers, and high-grade serous ovarian carcinoma (HGSOC) accounts for 70–80% of ovarian cancer deaths during several decades [1]. Thus, extraordinary studies are required to identify potential therapeutic targets for HGSOC management. We comprehensively synthesize local and public datasets about HGSOC development and progression to discover more about potential therapeutic targets.

Aberrant cholesterol metabolism represented the major characteristics of cancer in clinics, and cholesterol and its metabolites (precursors and derivatives) were major determinants by modulating cell signaling events governing the hallmarks of cancer [2]. Squalene epoxidase (SQLE), a famous rate-limiting enzyme in cholesterol synthesis, was degraded by excess cholesterol and functioned in converting squalene to 2,3(S)-monooxidosqualene [3]. The sterol regulatory element-binding protein (SREBP) and liver X receptors (LXRs) represented important mechanisms by which intracellular cholesterol regulates SQLE expression [4, 5]. In addition, The p53-mediated transcriptional regulation and E2 ubiquitin-conjugating enzymes/E3 ligase-mediated ubiquitin-protease system played leading roles in SQLE mRNA levels and protein degradation [6–8]. The SQLE-mediated metabolic reprogramming is related to its potential oncogenic role, but it is not the only mechanism being looked at [9]. Liu et al. [10] demonstrated that SQLE regulated breast cancer stem cell (BCSCs) stemness. Cancer stem cells (CSCs) have been considered promising therapeutic targets for cancer therapy since it was first discovered in 1994 [11]. It was reported that tumor cell stemness contributes to HGSOC recurrence by enhancing the ability of tumor progression, drug-resistant metastasis, and self-renewal [12, 13]. Whether SQLE was a genetic driver for the stemness of HGSOC stem cells remained largely unknown.

The N^6^-methyladenosine (m^6^A) modification of RNA was widely involved in the metabolic recombination of tumor cells, thereby controlling tumor development [14]. The m^6^A methylation is involved in almost all the RNA cycle stages, including regulation of the transcription, maturation, translation, degradation, and stability of mRNA [15]. Here, we focused on SQLE to investigate how m^6^A acted on protein translation and subsequently regulated SQLE expression and HGSOC progression.

In this study, we found the abnormal expression of SQLE was regulated by the methyltransferase Wilms tumor 1-associated protein (WTAP), demethylases fat mass and obesity-associated (FTO), and the insulin-like growth factor 2 mRNA binding protein 3 (IGF2BP3). Here, SQLE is required for maintaining HGSOC stemness and plays a positive role in promoting both tumorigenesis and cholesterol synthesis in HGSOC. It implies that SQLE may serve as a novel therapeutic target in HGSOC.

## Results

### HGSOC included abnormal expression of regulators governing cellular cholesterol synthesis, uptake, export, and esterification, such as SQLE

**Fig. 1:**
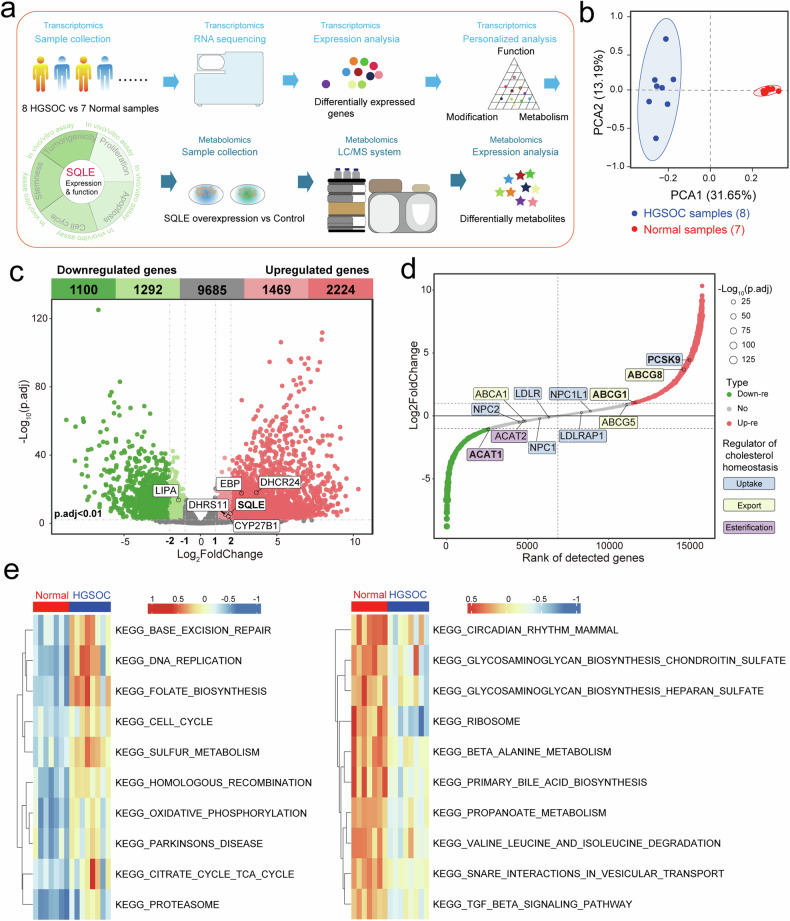
RNA sequencing was performed to identify potential therapeutic targets in high-grade serous ovarian cancer. **a** This study generated a data resource composed of RNA-seq profiles and metabonomics profiles. **b** The primary component analysis (PCA) of the data matrices was performed for the dimension reduction process. **c** The volcano map showed the number and distribution of differentially expressed genes (DEGs). These DEGs with |Log_2_FC | >1 and p.adj <0.05 were used for further analysis. There was significant difference in the expression of 5 metabolic enzymes in cholesterol biosynthesis between HGSOC samples and normal samples (p.adj <0.05). **d** The key factors governing cellular cholesterol uptake, export, and esterification were shown in the oncoplot. The red and green dots represented upregulated and downregulated genes, respectively. **e** Gene set variation analysis (GSVA) for RNA-sequencing data elucidated the molecular mechanisms of HGSOC patients and normal people.

We reported an RNA-seq transcriptomic study on HGSOC samples and normal ovarian tissue for defining the aberrant genes and dysregulated molecular pathways across HGSOC patients (Fig. 1a). The results of PCA suggested a high similarity between the samples in each group and a significant difference between the two groups (Fig. 1b). In the volcano plot, the x-axis displayed the log_2_FC values, and the y-axis corresponded to the -log_10_(adjusted. p-value). A total of 3693 upregulated genes and 2392 downregulated genes met the base threshold, among them, 2224 upregulated genes and 1100 downregulated genes had higher fold change (log_2_FC > 2 or log_2_FC < −2, respectively) than others. Furthermore, 6 out of 17 enzymes involved in the cholesterol synthesis met the threshold for screening DEGs and were labeled in this volcano plot, including SQLE (Fig. 1c). Except for synthesis, the dynamic balance between uptake, export, and esterification also controlled cellular cholesterol level [16].

The expression of 12 key factors governing those three parts cellular cholesterol uptake, export, and esterification was shown in the gene ranking oncoplot (Fig. 1d). Among them, the proprotein convertase subtilisin/kexin type 9 (PCSK9) that regulated cholesterol uptake [17], ABC subfamily G (ABCG) members 1 and 8 (ABCG1 and ABCG8) that regulated cholesterol efflux [18], and acyl-coenzyme A: cholesterol acyltransferase 1 (ACAT1) that regulated esterification [19], had abnormal expression in HGSOC compare to normal samples. In addition, the results of GSVA showed some pathways related to DNA synthesis and amino acid metabolism were enriched in the HGSOC samples (Fig. 1e). In the cascade mechanism, SQLE-induced upregulation of cholesterol synthesis may be the first step in the intracellular cholesterol homeostasis imbalance.

### Upregulation of SQLE was related to clinical features and poor prognosis of HGSOC

Based on the RNA-seq results, SQLE was an upregulated gene with log_2_FC equal to 1.96 and -Log_10_(p.adj) equal to 5.73. This trend was also found in 3 public datasets, such as TCGA/GTEx (426 ovarian serous cystadenocarcinoma samples and 88 normal ovarian samples), GSE69428 (10 HGSOC samples and 10 normal ovarian samples) and GSE124766 (8 HGSOC samples and 3 normal ovarian samples) (Supplementary Fig 1a). In addition, the qRT-PCR and WB assays of clinical samples showed the same results, indicating the high level of SQLE in the collected HGSOC sample (Supplementary Fig 1b–d). IHC staining of HGSOC samples with FIGO stage (I-IV) was used to detect SQLE expression. As shown in Supplementary Fig 1e, the SQLE protein was highly expressed in high-FIGO stages (III-IV) samples compared to those in low-stage samples (I-II). The chi-square test was used to analyze the relationship between SQLE expression and clinical factors of HGSOC patients. Results showed that the high expression of SQLE was positively related to the high FIGO stage (III and IV) and peritoneal metastasis (Supplementary Fig 1f). In addition, survival analysis showed that SQLE was associated with poor survival in OS, PFS, and PPS, suggesting its potential prognostic value in ovarian cancer and HGSOC patients (Supplementary Fig 1g, h). In samples with high FIGO stage, patients with high SQLE expression exhibited a median overall survival of only 40 months (median PFS 15.77 months; median PPS 36.67 months), while patients with low SQLE expression exhibited a median overall survival of 48 months (median PFS 17.77 months; median PPS 42.8 months). These data showed that high expression of SQLE was associated with poor prognosis and was an independent risk factor for survival.

### SQLE promoted the growth and migration of HGSOC cells in vitro

The CAOV-3 and OVCAR-3 cells, the most popular and suitable cell line models in HGSOC, did not differ in endogenous SQLE expression (Supplementary Fig 2a). The lentivirus-based pLVX-TetOne-Puro system and Tet-pLKO-Puro system were inducible expression systems and used for overexpressing or silencing SQLE under the treatment of Dox (Supplementary Fig 2b–d). To exclude an off-target effect of shSQLE, we generated shSQLE-resistant vectors carrying the synonymous mutational SQLE-shRNA target sequence to the pCDNA3.1-eGFP vector. As shown in Supplementary Fig 2e, HGSOC cells were mock transfected or transfected with the GFP-SQLE-mutation expression constructs, and then 48 h later, we determined the successful expression of the vector by fluorescence microscopy (Supplementary Fig 2f). Both SQLE mRNA and protein were silenced to below 30% in HGSOC cells with Dox-activated shRNA expression. In contrast, the SQLE expression was recovered in the rescue experiment using GFP-SQLE mutation (Supplementary Fig 2g, h). These findings suggested that shSQLE could specifically silence the SQLE gene.

**Fig. 2:**
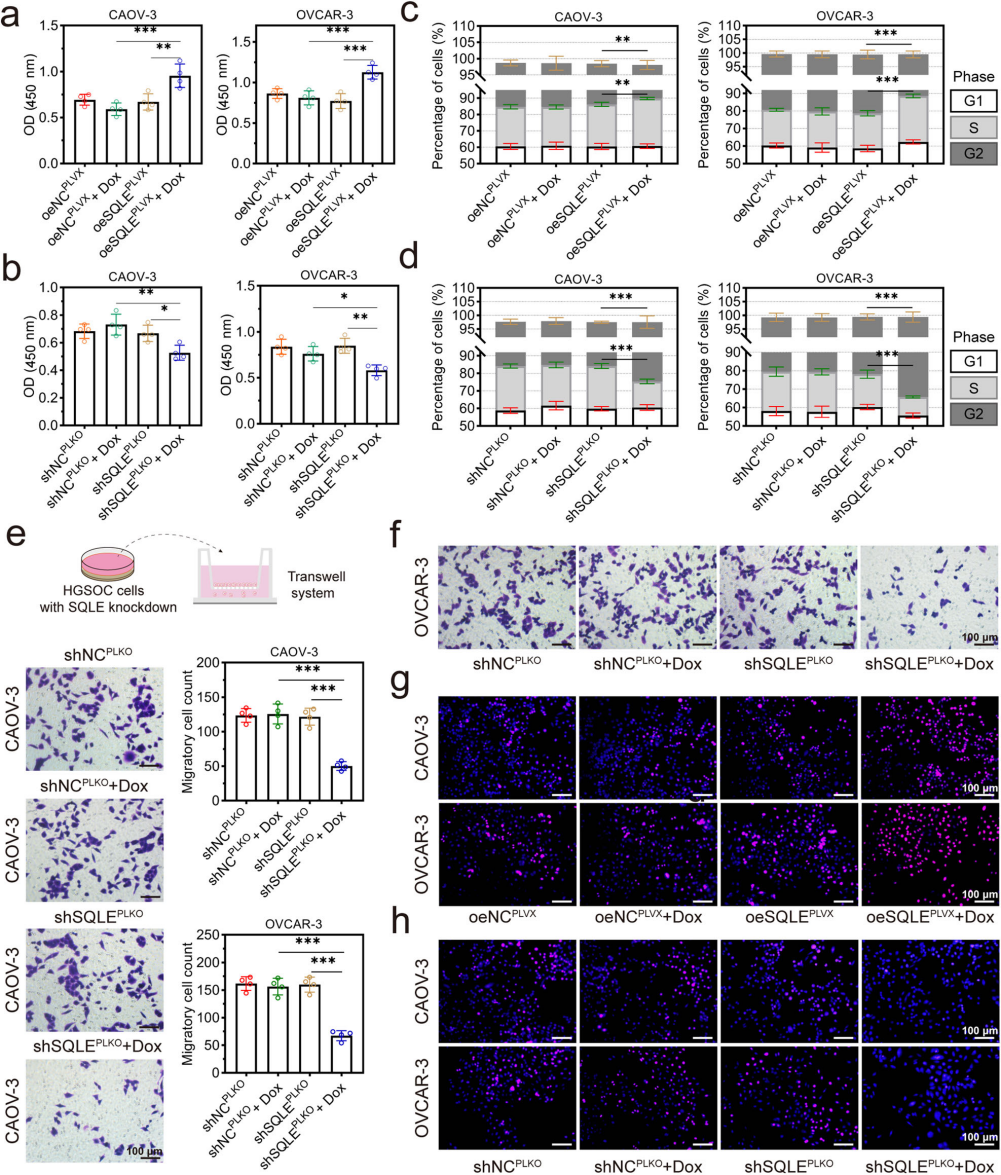
SQLE promoted the proliferation and migration of HGSOC cell lines. **a**, **b** The cell ability of CAOV-3 cells and OVCAR-3 cells was detected after transfecting with the pLVX-TetOne-Puro-SQLE vector or Tet-pLKO-Puro-shSQLE vector. **c**, **d** SQLE overexpression induced the increase of cell percentage in the S-phase and a decrease of the cell percentage in the G2-phase, whereas SQLE knockdown caused the opposite results. **e**, **f** Transwell assays detected the migration of CAOV-3 and OVCAR-3 cell with SQLE knockdown or not. Cell counts in lower chamber was observed under optical microscope with crystal violet staining. **g**, **h** The BrdU staining assays were used to detect the proliferating cells. Overexpression of SQLE increased the number of cells labeled with BrdU, whereas SQLE knockdown had the opposite results. Original magnifications for immunohistochemistry and crystal violet staining were 200×. *p < 0.05, **p < 0.01, and ***p < 0.001.

SQLE overexpression significantly increased cell viability, and SQLE knockdown decreased cell viability (Fig. 2a, b). SQLE overexpression induced an increase in the cell percentage of the S-phase and a decrease in the cell percentage of the G2-phase (Fig. 2c). SQLE knockdown had the opposite effect (Fig. 2d). The transwell assay showed that the number of migrating cells was significantly decreased after SQLE knockdown, suggesting that SQLE may play a critical role in the migration of HGSOC (Fig. 2e). In addition, BrdU-positive cells with red fluorescence were increased by SQLE overexpression and decreased by SQLE knockdown, suggesting the proliferation of new cells was regulated by SQLE level (Fig. 2f and Supplementary Fig 3a, b).

**Fig. 3:**
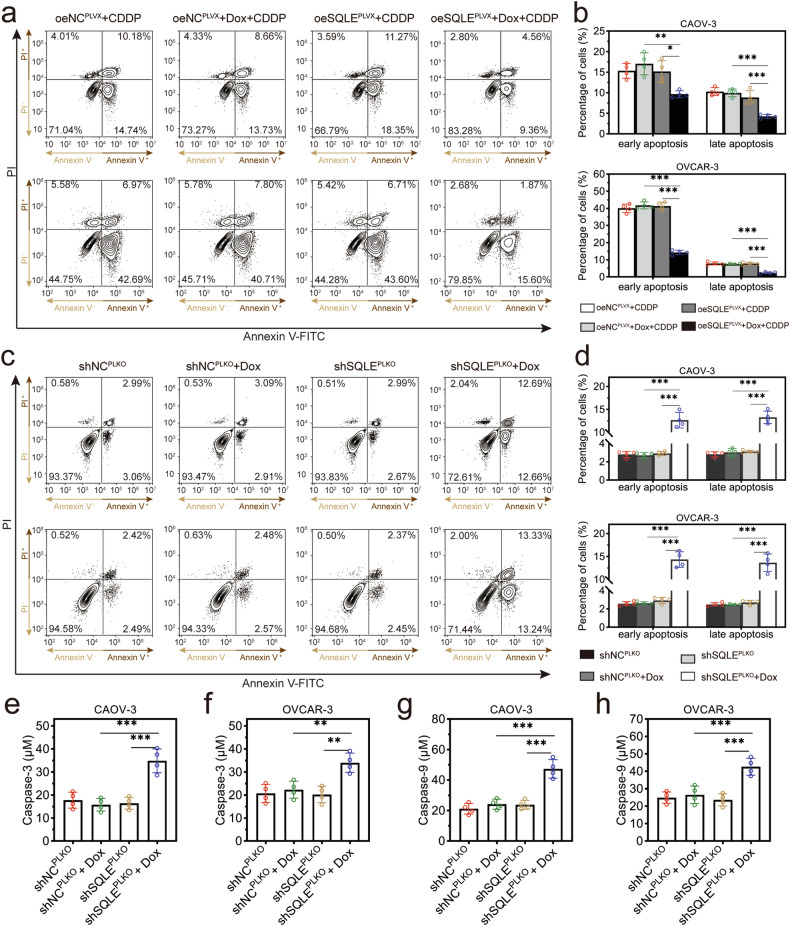
SQLE inhibited the HGSOC cell apoptosis. **a**, **b** Flow cytometry assays detected the cell apoptosis after SQLE overexpression and cisplatin (CDDP) treatment. **c**, **d** Flow cytometry assays detected the cell apoptosis after SQLE knockdown. Representative bivariate contour plots were shown, among them, Annexin V-positive and PI-positive cells were seen as late apoptosis, Annexin V-positive and PI-negative cells were seen as early apoptosis. **e**, **f** The levels of caspase-3 in CAOV-3 and OVCAR-3 cells with SQLE knockdown were detected. **g**, **h** The levels of caspase-9 in CAOV-3 and OVCAR-3 cells with SQLE knockdown were detected. *p < 0.05, **p < 0.01, and ***p < 0.001.

The cisplatin (CDDP) was used as an apoptotic stimulant when SQLE overexpression and flow cytometry assays showed significant decreases in the early apoptosis and late apoptosis levels after SQLE overexpression (Fig. 3a, b). SQLE knockdown significantly increased cell apoptosis compared with non-DOX-treated cells (Fig. 3c, d). The gating strategy for flow cytometry analysis was shown in Supplementary Fig 4a, b. The activities of caspase 3 and caspase 9 were enhanced by SQLE knockdown (Fig. 3e–h) and inhibited by SQLE overexpression (Supplementary Fig 5a–d). These results indicated that the upregulated SQLE contributed to the growth and migration of HGSOC cells.

### SQLE overexpression increased the stemness of OCSCs

**Fig. 4:**
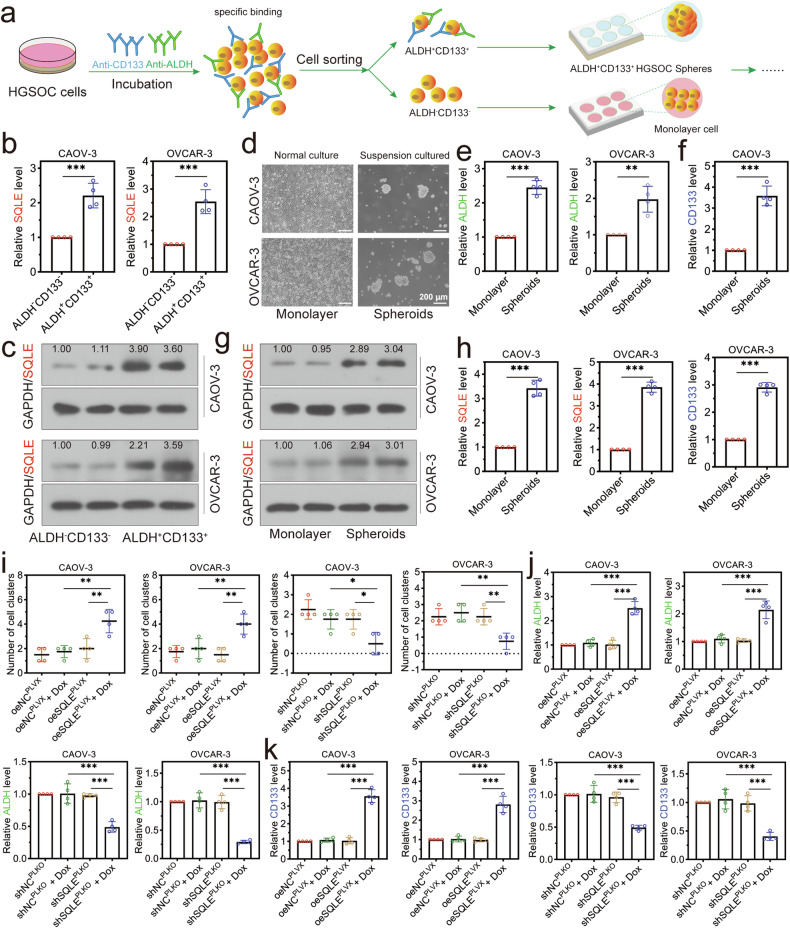
SQLE increased the sphere-forming potential of HGSOC cells. **a** Procedures for the stem cell screening and tumor sphere culture assay. **b**, **c** The SQLE level was higher in ALDH^+^CD133^+^ cells than in ALDH^-^CD133^-^ cells. **d** Sphere-formation assay analysis of sphere-forming capacities of ALDH^+^CD133^+^ cells. The typical pictures of monolayer cells and tumorspheres were shown. The magnification of the pictures was 100×. **e**, **f** The qRT-PCR analysis of ALDH expression and CD133 expression in 2D cultured monolayer cell and tumor spheres. **g**, **h** The SQLE highly expressed in the tumor spheres than in monolayer cells. **i** ALDH^+^CD133^+^ cells with SQLE overexpression exhibited significantly higher sphere-forming capacity, and SQLE knockdown had the opposite effect. **j**, **k** SQLE enhances the expression of stemness markers ALDH and CD133. *p < 0.05, **p < 0.01, and ***p < 0.001.

According to the procedure in Fig. 4a, whether the SQLE level influenced the stem-cell features of HGSOC cells was further explored. We first confirmed the expression of SQLE in the flow-sorted ALDH^+^CD133^+^ and ALDH^–^CD133^–^ cells from CAOV-3 or OVCAR-3 cells. The SQLE had higher expression at mRNA and protein levels in ALDH^+^CD133^+^ cells than ALDH^–^CD133^–^ cells (Fig. 4b, c). As shown in Fig. 4d, the spheres derived from CAOV-3 and OVCAR-3 cells could be observed after 14 days. The tumorspheres had high expression of ALDH and CD133, which suggested that culturing HGSOC cells under non-adherent conditions resulted in the formation of spheroids highly enriched in tumor cells with stem-like properties (Fig. 4e, f). We found a higher expression of SQLE in tumor spheres than in 2D-cultured monolayer cells (Fig. 4h). To further investigate whether the SQLE levels affected the stemness of tumorspheres, we analyzed the formation of tumorspheres. As presented in Fig. 4i, the numbers of tumorspheres formed specifically by CAOV-3 and OVCAR-3 cells were significantly increased by SQLE overexpression or decreased by SQLE knockdown, which suggested SQLE was required for HGSOC sphere formation. The typical pictures of tumorspheres were shown in Supplementary Fig 5e, f. Additionally, SQLE overexpression significantly increased mRNA expression levels of stemness marker ALDH and CD133 in tumorspheres formed specifically by CAOV-3 and OVCAR-3 cells. SQLE knockdown had opposite effects (Fig. 4j, k). These data indicated that SQLE increased the stemness of HGSOC cells and suggested an increased population of CSCs (ALDH^+^CD133^+^ cells) in these conditions.

The cell proliferation and stemness phenotype of HGSOC cells were tested further to comprehensively validate our findings and exclude the possibility of an off-target effect. As shown in Supplementary Fig 6a, the SQLE knockdown-induced loss of cell viability was rescued by overexpression of shRNA-resistant SQLE. In addition, the inhibited stemness phenotype resulting from SQLE knockdown was rescued by the expression of shRNA-resistant SQLE, reflecting in the recovered sphere formation ability and expression of stemness markers (Supplementary Fig 6b–e). SQLE knockdown phenotype was rescued by expression of shRNA-resistant mutant SQLE. Together, these experiments indicate that the key phenotype observed in SQLE knockdown cells is not caused by an off-target effect.

### SQLE enhanced the tumorigenicity of HGSOC cells

**Fig. 5:**
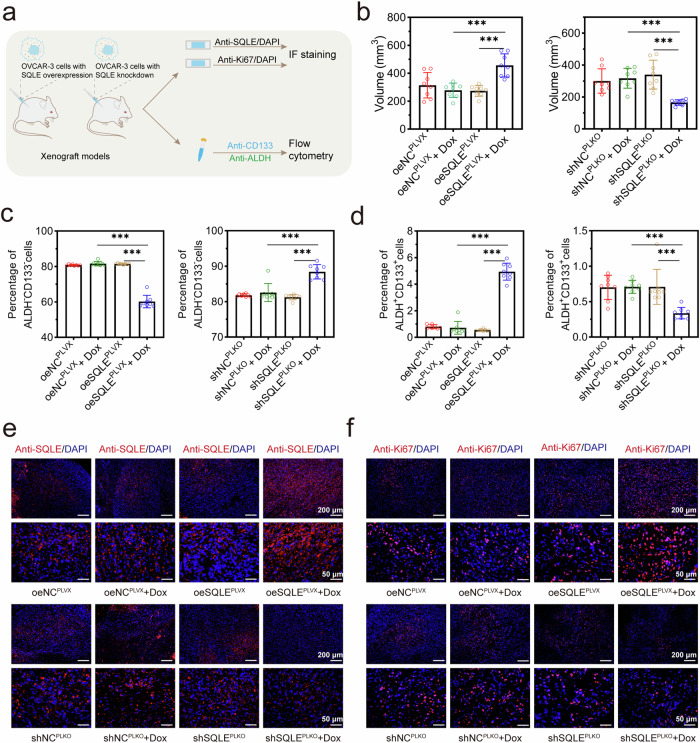
SQLE enhanced tumorigenic potential of HGSOC cells in vivo. **a** The tumor-bearing tissues were collected, and the stemness and malignancy of the tumor were detected. **b** The volume of subcutaneous tumors was detected after injecting OVCAR-3 cells with SQLE knockdown or SQLE overexpression. **c** The percentage of ALDH^-^CD133^-^ cells in tumor-bearing tissue was decreased by SQLE overexpression but increased by SQLE knockdown. **d** The percentage of ALDH^+^CD133^+^ cells in tumor-bearing tissue was increased by SQLE overexpression but decreased by SQLE knockdown. The expression of SQLE (**e**) and Ki67 (**f**) in tumor samples was analyzed. Cell nucleus were observed by DAPI. Original magnifications for immunohistochemistry were 100× and 400×. *p < 0.05, **p < 0.01, and ***p < 0.001.

To determine the impact of abnormal SQLE expression on the tumor-initiating potential of OVCAR-3 cells, we constructed the xenotransplantation in immunodeficient mice of the OVCAR-3 cells with SQLE overexpression and knockdown, respectively (Fig. 5a). Tumors derived from shSQLE-transduced and DOX-treated OVCAR-3 cells displayed smaller tumor volumes at the endpoint compared with xenografts from the mice without DOX treatment (Fig. 5b). The collected fresh xenografts were dissociated into the single-cell suspension for flow cytometry analysis. The gating strategy was shown in Supplementary Fig 5g. The results showed that the CSCs (ALDH^+^CD133^+^ cells) population was remarkably increased in the oeSQLE^PLVX^ + DOX group compared with in the oeSQLE^PLVX^ group, and decreased in the shSQLE^PLKO^ + DOX group compared with in the shSQLE^PLKO^ group (Fig. 5c). The ALDH^-^CD133^-^ population had corresponding changes (Fig. 5d). The IF staining of tumor xenografts verified the SQLE expression (Fig. 5e). In addition, SQLE overexpression increased the Ki67 protein level of tumor xenografts, suggesting the grade malignancy of the tumors (Fig. 5f). These data confirmed that SQLE enhanced the tumorigenic ability of HGSOC cells and increased HGSOC CSCs.

### SQLE overexpression induced metabolism reprogramming, especially altered cholesterol metabolism in HGSOC

The metabolomics was used to study the key metabolic pathway changes induced by SQLE overexpression. The detected features in positive and negative modes were analyzed using the PCA and OPLS-DA models. These results clearly distinguished SQLE overexpression and control groups in positive and negative modes (Supplementary Fig 5h–k). In the metabolomic profiling through secondary identification, we found 250 metabolites, among them, 49 upregulated metabolites (VIP ≥ 1 & p.adj < 0.05 & FC > 1.2) and 41 downregulated metabolites (VIP ≥ 1 & p.adj < 0.05 & FC < 0.8) were identified by statistical analysis.

**Fig. 6:**
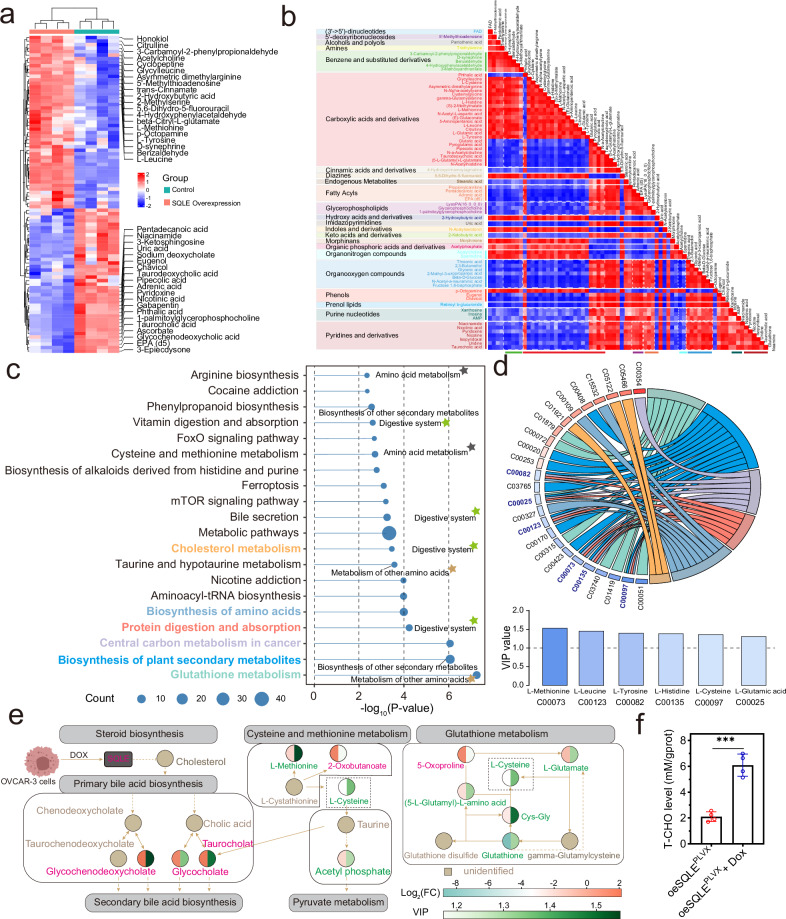
SQLE overexpression promoted cholesterol metabolism. **a** Heatmap of differentially expressed metabolites (DEMs). **b** Correlation analysis between differential metabolites. **c** These DEMs were involved in many metabolism pathways. **d** The chord diagram displayed the correspondence of genes and pathways. The VIP values of shared DEMs were shown in a histogram. **e** The DEMs were involved in cholesterol production, cholesterol metabolism, and glutathione metabolism. **f** SQLE overexpression increased the level of total cholesterol. *p < 0.05, **p < 0.01, and ***p < 0.001.

As shown in Fig. 6a, the clustering heat map showed the expression of 90 DEMs in each sample and the names of the top 20 upregulated and downregulated DEMs. Metabolomics profiling annotation was performed using database searching. These DEMs were divided into 23 categories, among them, 23 DEMs belonged to carboxylic acids and derivatives, and 7 DEMs belonged to organooxygen compounds (Fig. 6b). The correlation analysis showed the DEMs that belonged to the benzene and substituted derivatives class negatively related to those DEMs in the pyridines and derivatives class, organooxygen compounds class, and part carboxylic acids and derivatives class. These DEMs in the glycerophospholipids class had significant positive correlations with DEMs in the pyridines and derivatives class and organooxygen compounds class (Fig. 6b). To explore the identities and functions of these DEMs, we showed the top 20 metabolic pathways enriched by DEMs in Fig. 6c. SQLE overexpression induced significant changes in cholesterol metabolism, biosynthesis of amino acids, protein digestion and absorption, central carbon metabolism in cancer, biosynthesis of plant secondary metabolites, and glutathione metabolism pathways (Fig. 6c). A chord diagram exhibited the relationship between DEMs and the above pathways. The shared DEMs, including L-methionine, L-Leucine, L-Tyrosine, L-histidine, L-Cysteine, and L-glutamic acid, had high VIP values (Fig. 6d). The brief map between the main metabolic pathways was displayed based on the KEGG database (Fig. 6e). The downstream pathway of cholesterol, the primary bile acid biosynthesis pathway, contained a series of changes. L-cysteine was a key metabolite, connecting the altered metabolic pathway after SQLE overexpression (Fig. 6e). As shown in Fig. 6f, SQLE overexpression enhanced the level of total cholesterol. Metabolomics revealed that the SQLE-induced reprogrammed signaling pathways regulated cancer development.

### Identified SQLE as a targeted regulation gene by WTAP/FTO-mediated m^6^A modification

Three volcano maps showed the expression of the m^6^A regulator, including two differently expressed “writers” (METTL14 and METTL16), one differently expressed “eraser” (FTO), four differently expressed “readers” (IGF2BP1/2/3, and YTHDC1) in the RNA-Seq results (Supplementary Fig 7a). In addition, we found that these differently expressed m^6^A regulators were highly associated with many DEGs in clinical samples, suggesting the extensive and complex m^6^A methylation across the transcriptome. To detail, 2652 DEGs had a high correlation with METTL14, METTL6, FTO, and YTHDC1 at the same time. 337 DEGs associated with METTL14, METTL6, FTO, YTHDC1, IGF2BP2, and IGF2BP3 simultaneously (Supplementary Fig 7b). The survival analysis revealed the WTAP had prognostic value in ovarian cancer samples with all stages and ovarian cancer samples with high stages (III and IV) (Supplementary Fig 7c).

To test our speculation, the relationship between SQLE, WTAP, and FTO was further discussed. The qRT-PCR assays revealed the upregulation of WTAP and downregulation of FTO in clinical HGSOC samples (Supplementary Fig 7d, e). We found a positive correlation between WTAP and SQLE (*r* = 0.76), and a negative correlation between FTO and SQLE (*r* = −0.74) (Supplementary Fig 7f). Based on the above findings, we further designed the function gain-/loos- assay in OVCAR-3 cells. The efficiency of FTO and WTAP overexpression or knockdown was verified by qRT-PCR (Supplementary Fig 7g, h). The WTAP overexpression enhanced the SQLE level (Supplementary Fig 7i). The demethylase FTO overexpression inhibited the SQLE expression, and FTO knockdown increased the SQLE expression (Supplementary Fig 7j). Collectively, these data also verified that WTAP and FTO oppositely regulated the expression of SQLE. Furthermore, SQLE could be a potential downstream target of the WTAP/FTO axis in HGSOC.

### The OCSLCs had abundant m^6^A modification of SQLE

**Fig. 7:**
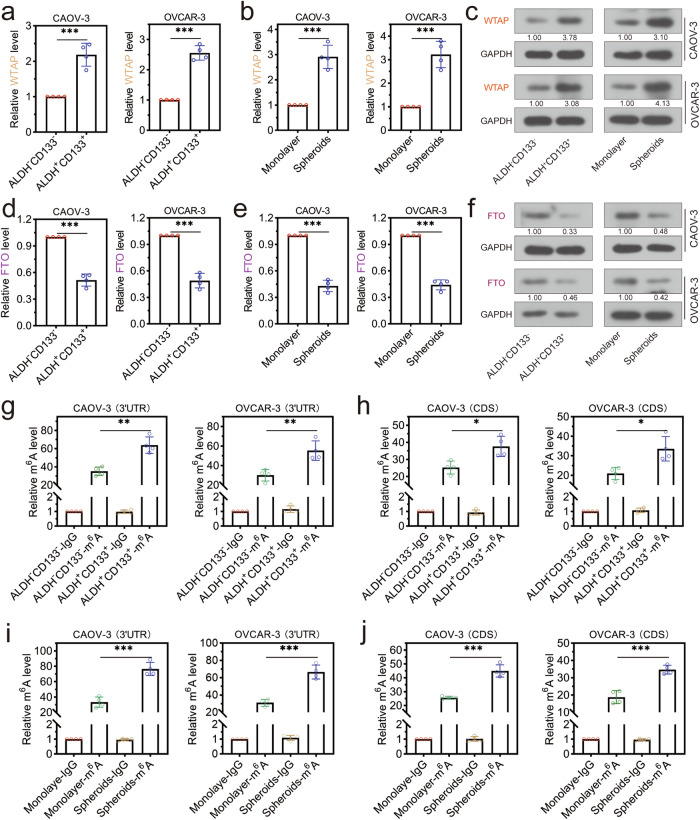
The m^6^A level of SQLE was mediated by methyltransferase WTAP and demethylase FTO. **a**–**c** The WTAP was highly expressed in ALDH^+^CD133^+^ cells and tumor spheres than ALDH^-^CD133^-^ cells and monolayer cells, respectively. **d**–**f** The FTO was lowly expressed in ALDH^+^CD133^+^ cells and tumor spheres than ALDH^-^CD133^-^ cells and monolayer cells, respectively. **g**, **h** The ALDH^+^CD133^+^ cells had higher levels of m^6^A modifications at the 3’UTR and CDS regions of SQLE mRNA than in the ALDH^-^CD133^-^ cells. **i**, **j** The tumorspheres had higher levels of m^6^A modifications at the 3′UTR and CDS regions of SQLE mRNA than in the monolayer cells. *p < 0.05, **p < 0.01, and ***p < 0.001.

To further explore the role of the m^6^A modification in HGSOC, we found the differentially expressed m^6^A regulators in OCSLCs and their functions. The ALDH^+^CD133^+^ cells had higher WTAP levels and lower FTO levels than the ALDH^-^CD133^-^ cells (Fig. 7a, d). Similarly, the tumorspheres had higher WTAP levels and lower FTO levels than adherent cultured monolayer cells (Fig. 7b, e). WB assays verified the high expression of WTAP and low expression of FTO proteins (Fig. 7c, f). Furthermore, we found a higher level of m^6^A modification in ALDH^+^CD133^+^ cells than in ALDH^-^CD133^-^ cells, whether in 3′UTR or CDS regions (Fig. 7g, h). In addition, the tumorsphere derived from CAOV-3 or OVCAR-3 cells also had a higher level of on the 3′UTR and CDS regions of SQLE mRNA (Fig. 7I, j). These data suggested that HGSOC CSCs and tumorspheres had high m^6^A levels of SQLE mRNA, which was related to WTAP and FTO expression.

### WTAP overexpression and FTO knockdown enhanced SQLE mRNA stability via an m^6^A-IGF2BP3-dependent pathway

**Fig. 8:**
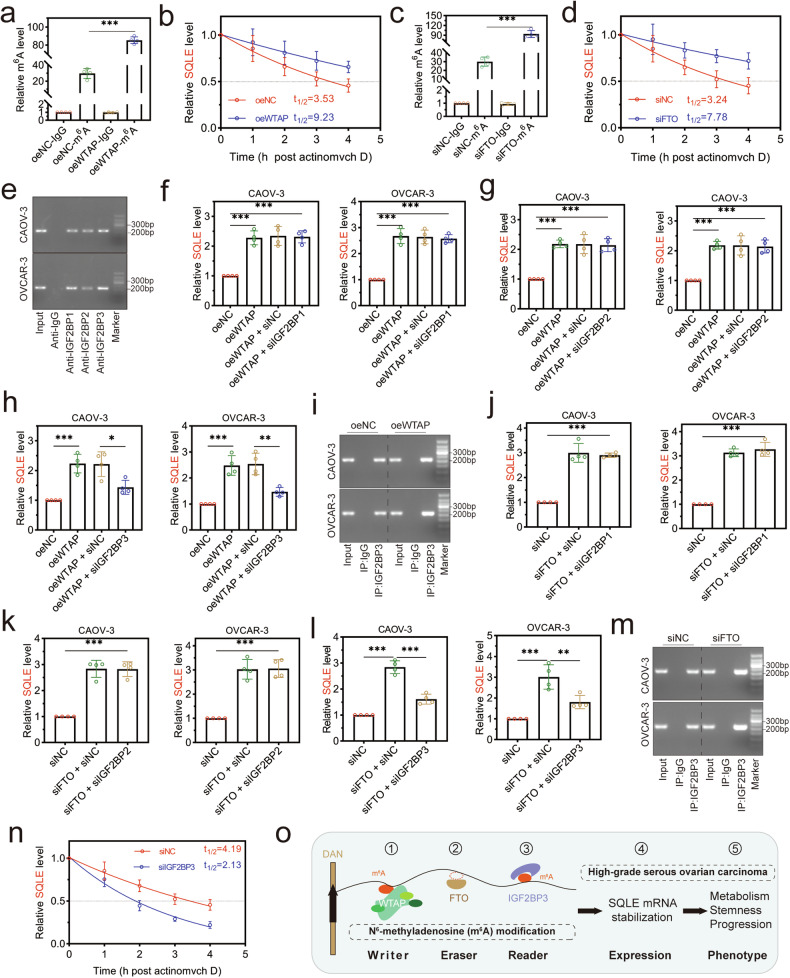
Methylated reading protein IGF2BP3 enhanced the stability of SQLE mRNA in a m^6^A-dependent manner. **a** The WTAP positively regulated the m^6^A modification of SQLE mRNA. **b** Overexpression of WTAP enhanced the half-life of SQLE mRNA. **c** The FTO negatively regulated the m^6^A modification of SQLE mRNA. **d** Knockdown of FTO enhanced the half-life of SQLE mRNA. **e** RIP assays showed the combination of IGF2BP1/2/3 with SQLE mRNA. **f**, **g** Knockdown IGF2BP1/2 did not reverse the WTAP-induced m^6^A level of SQLE mRNA. **h** Knockdown IGF2BP3 reversed the high m^6^A level of SQLE mRNA that was caused by WTAP overexpression. **i** RIP assays showed that the interaction of IGF2BP3 with SQLE mRNA was enhanced in cells with WTAP overexpression compared to the control group. **j**, **k** Knockdown IGF2BP1/2 did not reverse the FTO knockdown-induced high m^6^A level of SQLE mRNA. **l** Knockdown IGF2BP3 reversed the high m^6^A level of SQLE mRNA that was caused by FTO knockdown. **m** RIP assays showed that the interaction of IGF2BP3 with SQLE mRNA was enhanced in cells with FTO knockdown compared to the control group. **n** Knockdown of IGF2BP3 decreased the half-life of SQLE mRNA. **o** The m^6^A modification of SQLE mRNA was installed by the m^6^A methyltransferases WTAP, reverted by the demethylases FTO, and recognized by m^6^A binding proteins IGF2BP3. *p < 0.05, **p < 0.01, and ***p < 0.001.

It was time to explore the precise mechanism for the m^6^A modifications of SQLE mRNA. Firstly, WTAP overexpression enhanced the m^6^A methylation level of SQLE and extended the half-life of SQLE mRNA (Fig. 8a, b). Similarly, FTO knockdown also enhanced the m^6^A methylation level of SQLE and increased the half-life of SQLE mRNA (Fig. 8c, d). These results proved that the m^6^A modification of SQLE was installed by methyltransferases WTAP and reverted by demethylases FTO. To confirm whether IGF2BP1/2/3 were potential “readers” in SQLE m^6^A methylation, the direct binding interactions between IGF2BP1/2/3 and SQLE mRNA were observed in RIP assays compared with IgG (Fig. 8e). The combined effects of WTAP/FTO and IGF2BP1/2/3 were analyzed step by step. Firstly, a cross-check of IGF2BP1/2/3 expression was performed to verify the specificity of siIGF2BP1/2/3. As shown in Supplementary Fig 8a, only the cells with siIGF2BP1 transfection showed a significant decrease in IGF2BP1 mRNA expression, and did not affect the IGF2BP2 and IGF2BP3 expression. The cells with siIGF2BP2 or siIGF2BP3 transfection also showed such highly specific results. Thus, this set of siRNA was used in follow-up experiments. The results from qRT-PCR analysis showed that overexpression of WTAP led to a substantial increase in SQLE expression, while IGF2BP1/2 knockdown did not influence this effect, and IGF2BP3 knockdown reversed this effect (Fig. 8f–h). Meanwhile, the WTAP overexpression enhanced IGF2BP3 binding with SQLE mRNA (Fig. 8i). In addition, IGF2BP3 knockdown also reversed the increased SQLE expression and the interaction between FTO and SQLE mRNA induced by FTO knockdown (Fig. 8g–i).

The relationship between IGF2BP3 and SQLE needed more evidence. Firstly, the effects of IGF2BP3 on the stability of SQLE mRNA and SQLE-induced phenotype were detected. The half-life of SQLE was more than 4 h when siNC transfection, and IGF2BP3 knockdown caused it to shorten by half (Fig. 8n). To explore whether IGF2BP3 contributed to the functional phenotype of the cell induced by SQLE, IGF2BP3 was silenced by shRNA that targeted the same sequence as siIGF2BP3 in cells with SQLE overexpression or not (Supplementary Fig 8b). IGF2BP3 knockdown induced the decrease of SQLE expression, which was reversed after Dox-activating the overexpression vectors (Supplementary Fig 8c). Cell viability confirmed that the inhibition of cell proliferation induced by IGF2BP3 knockdown was recovered by SQLE overexpression. In addition, the sphere formation assay presented that IGF2BP3 knockdown resulted in the formation of significantly fewer and smaller spheres compared with those in the shNC group (Supplementary Fig 8e, f). In addition, SQLE could successfully reverse the stemness markers suppression caused by IGF2BP3 knockdown (Supplementary Fig 8g, h). These findings identified that IGF2BP3/SQLE formed a functional axis in regulating cell growth and stemness phenotype in HGSOC.

Taken together, these results demonstrated that m^6^A methyltransferases WTAP, demethylases FTO, and methyl-binding proteins IGF2BP3 regulated the m^6^A methylation of SQLE collaboratively (Fig. 8o).

## Discussion

Sufficient evidence supported that tumor-specific CSCs existed in ovarian cancer, which were functionally similar to normal stem cells. The OCSCs could be identified according to the differential expression of cell surface markers and differential biochemical properties, typically CD133 and ALDH [20]. However, the drivers of CD133^+^ALDH^+^ cells and OCSCs stemness remain largely unclear. In the current study, we selected genes with prognostic value via RNA-Seq and confirmed that SQLE, a key enzyme in cholesterol metabolism, was highly expressed in HGSOC. In addition, we isolated the CD133^+^ALDH^+^ subpopulation and cultured tumorspheres to explore the influence of SQLE on the stemness of HGSOC. Furthermore, we demonstrated that the m^6^A modification of SQLE was regulated by the WTAP/FTO/IGF2BP3 axis, inducing the upregulation of SQLE, thereby promoting the HGSOC stemness and progression.

The expression of SQLE was regulated by multiple mechanisms in cancer, including microRNA, long non-coding RNA, ubiquitin ligases, and transcription factors [7, 21]. The upregulation of SQLE was associated with the poor prognosis of breast cancer, colorectal cancer, and pancreatic adenocarcinoma [22–24]. These reports all identified SQLE as a valuable prognostic marker in cancer development. In this study, a close connection between the high expression of SQLE with the high FIGO stage or peritoneal metastasis was shown. It was known that the early onset of peritoneal dissemination was an important difference between HGSOC and low-grade serous ovarian cancer (LGSOC) [25]. SQLE-high HGSOC patients show significantly better OS, and may have 8 months of beneficial survival. The clinical value of SQLE needs to be verified by more experiments. If it can be turned into a targeted drug, like PARP inhibitors, more patients may benefit from the new treatment [26]. There is currently no clinical use of SQLE for cancer treatment, but a clinical trial identified that SQLE was related to the clinical response to terbinafine in patients with tinea corporis/cruris [27]. Therefore, the development of drugs targeting the SQLE gene may have a broad prospect in clinical therapy.

The expression of SQLE mainly depended on its m^6^A modification in this study. We paid more attention to the m^6^A modification of SQLE based on predicted results that existed credible m^6^A sites. Among them, *Yang* et al. reported a high expression and prognostic value of m^6^A regulator WTAP in HGSOC, and *Xu* et al. reported a low expression of FTO in ovarian serous carcinoma [28, 29]. These reports were consistent with our results. Functionally, the upregulated WTAP recruited and interacted with METTL3 and METTL14 to increase the formation of methyltransferase complexes, known as “writers”. Meanwhile, FTO functioned as demethylases for the reverse effect, catalyzing the demethylation of m^6^A [30]. Indeed, METTL3, METTL14, and FTO have been clarified to affect the progression of ovarian cancer in a m^6^A-dependent manner [31–33]. In this study, we speculated that WTAP and FTO participated in HGSOC progression via opposite regulating m^6^A modification of SQLE mRNA. Notably, reader proteins interpreted m^6^A modification and regulated mRNA fate, and IGF2BPs-dependent m^6^A post-transcriptional expression played oncogenic roles in cancers via recognizing the consensus GG(m6A)C sequence [34]. Chen et al. reported that over 5000 mRNAs were targeted by at least one IGF2BPs protein [35]. We found the combination of SQLE mRNA with IGF2BPs via RIP assay and the enhancement of SQLE mRNA stability, whereas rescue assay showed only IGF2BP3 knockdown reversed the effect of WTAP overexpression or FTO knockdown on the SQLE expression. This specific recognition effect of IGF2BPs, which was similar to our results, was reported in the study of m^6^A methylated EphA2 and VEGFA [36]. Despite the high similarity in their functional N-terminal RNA recognition motifs (RRM) and C-terminal hnRNP K homology (KH) domains, IGF2BPs exhibited distinct RNA-binding properties, thereby inducing different target mRNA fate [37]. In addition, IGF2BPs not only maintained target mRNA stability, but directed the localization of target mRNA or transient storage during cellular stress [38–41]. In contrast to IGF2BP3-SQLE mRNA complexes, which had been found to contribute to the stability of SQLE mRNA, whether IGF2BP1-SQLE mRNA complexes and IGF2BP2-SQLE mRNA complexes are involved in HGSOC development through different mechanisms needs further study.

SQLE-mediated cholesterol homeostasis participated in tumor growth through various factors, mainly increased tumorigenicity and metastasis of cancers [42]. SQLE-mediated cholesterol increase in breast tumors promotes CSC formation and stemness maintenance [10]. In this study, the bile acids biosynthesis pathway, a major secondary pathway of cholesterol, was activated by SQLE overexpression in HGSOC cells. These were three upregulated metabolites of cholesterol, including glycocholic acid (GC), taurocholic acid (TCA), and glycochenodeoxycholic acid (GCDC). Detailedly, GC has been identified as a potential biomarker candidate for ovarian cancer and was associated with a poor prognosis of hepatocellular carcinoma [43, 44]. TCA had the potential of carcinogen and tumor-promoter [45]. Zhu et al. demonstrated that upregulated GCDC induced stemness of hepatocellular carcinoma (HCC) cells [46]. Based on these findings, we speculated that SQLE regulated cholesterol synthesis and metabolism, thus contributing to the maintenance of HGSOC stemness. Moreover, we found that glutathione metabolism and its related pathways were involved in the stemness and progression of HGSOC. Wang et al. indicated that Frizzled-7 (FZD7) enhanced stemness features by driving the upregulation of glutathione metabolism pathways in ovarian cancer [47]. Especially, L-cysteine was a bridge that connected the cholesterol metabolism pathway and glutathione metabolism pathway in this study. Cysteine maintained tumor stemness by increasing the expression of c-Myc in cancer stem cells [48]. Together, our results reveal that SQLE maintained HGSOC stemness through reprogrammed metabolism of cholesterol and glutathione.

In conclusion, we comprehensively estimated the m^6^A modification of SQLE and corresponding proliferation, apoptosis, metabolism, and stemness characteristics in HGSOC. In clinical, terbinafine, an SQLE inhibitor, reduced levels of prostate-specific antigen in late-stage prostate cancer patients [9]. Based on the above findings, SQLE will be a valuable target in the treatment of HGSOC.

## Materials and methods

### Patients’ sample

A total of 77 ovarian cancer samples and 40 normal ovarian tissue were collected from Shengjing Hospital of China Medical University (Liaoning, China). Written informed consent was obtained from all patients according to the policies of the committee. The detailed clinicopathological features of cancer samples, such as age, histological type, histopathological grade, lymph node metastasis, distant metastasis, peritoneal metastasis, and the International Federation of Gynecology and Obstetrics (FIGO) stage were available. Of the 77 ovarian cancer samples, there were FIGO stage I (*n* = 8), FIGO stage II (*n* = 5), FIGO stage III (*n* = 40), and FIGO stage IV (*n* = 24).

### RNA-sequencing, function enrichment analysis, and survival analysis

Total RNA was extracted from 8 HGSOC samples and 7 normal ovarian samples using TRIzol reagent. The integrity and concentration of total RNA were detected by Agilent 2100 bioanalyzer (Agilent, US). Then the poly(A) RNA was fragmented into small pieces and was reverse-transcribed to create the cDNA by SuperScript™ II Reverse Transcriptase (Invitrogen, US). Sequencing was performed at the Lianchuan biotech company on an Illumina Novaseq™ 6000 (LC-Bio Technology Co., Ltd., China) in 2 × 150 bp paired-end sequencing. The cleaned sequencing reads were then mapped to the human reference genome (build hg. 19). FeatureCounts software was used to count reads mapped to RefSeq genes, and the transcription abundance for each gene was calculated by Cuffdiff 2 Software. The fragments per kilobase of exon model per million fragments mapped (FPKM) values were analyzed based on the principle component analysis (PCA). With threshold value of |Log_2_Flod Change (FC) | > 1 and *p* < 0.05, We screened the differentially expressed genes (DEGs) for further function enrichment analysis. The gene set variation analysis (GSVA) of all gene expression profiles was performed using the R package GSVA version 1.46.0. Kaplan-Meier and gene expression correlation analyses Hazardous ratios (HRs) for indicated gene panels and tumor cohorts of serous ovarian carcinoma and HGSOC were determined by the Kaplan-Meier (KM) plotter (http://kmplot.com/analysis/index.php?p=background) online tool using best cutoff analyses and the multigene classifier.

### Enzymes and regulators involved in intracellular cholesterol homeostasis

Cholesterol metabolism could be divided into four parts, biosynthesis, uptake, export, and esterification, in dynamic balance [16]. The enzymes that regulated cholesterol synthesis were screened out from the steroid biosynthesis (or cholesterol biosynthesis) pathway in the Kyoto Encyclopedia of Genes and Genomes dataset (KEGG, https://www.genome.jp/kegg/) and the Small Molecule Pathway Database (SMPDB, http://www.smpdb.ca/). In addition, the main regulators in uptake, export, and esterification were collated from the literature, and mapped in the cholesterol metabolism pathway (KEGG, hsa04979) to determine its functionality. The expression of these factors was discussed based on the RNA-sequencing data.

### Quantitative real-time PCR analysis (qRT-PCR)

Total RNA samples were reverse transcribed to cDNA by reverse transcriptase. Gene expression was detected using SYBR Green reagent kits (Solarbio, China) in an ExicyclerTM 96 fluorescence ratio PCR instrument (Bioneer, Korea). Experimental procedures were as follows: 95 °C for 5 min, followed by 40 cycles of 95 °C for 10 s, 60 °C for 10 s, and 72 °C for 15 s. Primers were listed in supplementary Table 1. Relative expression levels were calculated after normalization to the reference gene glyceraldehyde-3-phosphate dehydrogenase (GAPDH) according to the 2^-ΔΔCt^ method.

### Western blotting

Protein samples were extracted from cells and tissues with RIPA buffer (Beyotime Biotechnology, China). An equal amount of protein was separated by sodium dodecyl sulfate-polyacrylamide gel electrophoresis (SDS-PAGE), after which it was transferred to PVDF membranes (Millipore, US). After blocking with 5% skimmed milk, the membranes were incubated with primary and secondary antibodies. The immunoblot was developed using a chemiluminescence (ECL) reagent (Beyotime Biotechnology, China). Antibody information was provided as supplementary Table 2.

### Immunohistochemistry (IHC) staining and semiquantitative analysis

After paraffin embedding, sectioning, dewaxing, antigen preparation, and serum blocking, samples were stained with SQLE antibody (Abclonal, China) and HRP-conjugated goat anti-rabbit second antibody (ThermoFisher, US). IHC staining was semi-quantitatively assessed based on stain intensity and the percentage of positive cells, which were scored independently by two pathologists. Stain intensity was categorized as 0 (i.e., negative), 1 (weakly positive), 2 (moderately positive), or 3 (strongly positive). The percentage of positive cells was categorized, as follows; 0 (i.e., <10%), 1 (10–20%), 2 (21–50%), or 3 ( < 50%). Final scores for each section were then calculated by multiplying stain intensity scores by stain percentages. Immunohistochemical scores that were great than the median of all scores were considered high expression, and less than the median of all scores were considered low expression.

### Cell Lines

The human ovarian cancer cell lines CAOV-3 and OVCAR-3 were bought from iCell biotechnology company (China). CAOV-3 cells were maintained in Dulbecco’s modified eagle medium (Servicebio, China) supplemented with 10% FBS (TianHang biotechnology company, China) at 37 °C and 5% CO_2_. OVCAR-3 cells were maintained in Roswell Park Memorial Institute-1640 medium (Solarbio, China) supplemented with 20% FBS at 37 °C and 5% CO_2_. All 2-D monolayer cultures (2-D), including monolayer cell culture, were maintained in the above standard medium.

### Spheroid formation assay

To generate spheroids, 5 × 10^3^ cells/well were seeded in 6-well Attachment Surface Polystyrene (Corning Costar, US). 3-D cultures were maintained in serum-free Dulbecco’s Modified Eagle Medium/Nutrient Mixture F-12 (DMEM-F12) medium (Biosharp, China) supplemented with 20 μg/L human recombinant epidermal growth factor (EGF) (GenScript, China), 10 μg/L basic fibroblast growth factor (bFGF) (GenScript, China), and 2% B27 supplement (Biosharp, China). After 14 days in culture, the tumorsphere were collected for further assays. The tumorspheres enriched with ovarian cancer stem cells (OCSCs) were used as a model for stemness study.

### Lentivirus infection and generation of the stable expressing SQLE

In this study, the lentivirus-mediated SQLE overexpression system: pLVX-TetOne-Puro-SQLE and lentivirus-mediated SQLE knockdown system: Tet-pLKO-Puro-shSQLE were purchased from Fenghui biotechnology company (China), which lentiviral vectors carried puromycin tags and doxycycline-responsive transactivator. Cells were seeded in 6-well culture plates, infected with lentivirus carrying inducible expression systems for 24 h, and then cultured in normal mediums for 24 h. Moreover, cells were further cultured in 1.5 μg/mL of puromycin (Macklin, China) to screen for cell lines that stabilize differential gene expression. In the treatment of 1 μg/mL doxycycline (Dox) for 72 h, the SQLE expressions were verified through qRT-PCR and WB assays. In addition, the siWTAP, siFTO, siIGF2BP1, siIGF2BP2, and siIGF2BP3 were synthesized by Jintuosi biological technology (China) and transfected into cells using Lipofectamine 3000 (Invitrogen, US) according to the manufacturer’s protocol. The sequences of shRNA and siRNA used in this study were listed in supplementary Table 3. In addition, we constructed GFP-based vectors carrying the CDS region of SQLE with 5 synonymous point mutations within shRNA target regions (named shSQLE-resistant vectors). These vectors were used to rescue the expression of SQLE-targeted genes and the corresponding phenotype change to prevent the generation of “off-target effects” resulting from possible identity between shRNA and abundant cellular mRNA transcripts [49].

### Cell ability and the caspase-3/9 activity

In total 2 × 10^3^ cells/well were seeded into 96-well cell culture plates and treated with or without Dox for 0, 12, 24, 48, and 72 h. Then 10 μL WST-1 solution was added and incubated for 4 h at 37 °C. The absorbance at 450 nm was measured on an automated microplate reader (BIOTEK, US).

The activity of Caspase-3 and Caspase-9 were assessed by the Caspase 3 activity assay kit and Caspase 9 activity assay kit (Beyotime, China). The activity of Caspase-3/9 was normalized to protein content that was detected by the Bradford protein assay kit (Beyotime, China).

### Flow cytometry analysis of cell cycle and apoptosis

The cell cycle test kit (KeyGEN, China) was used to detect the cell cycle of CAOV-3 and OVCAR-3 cells. Briefly, cells were harvested by trypsinization, centrifuged at 150 g, and fixed in 75% ice-cold ethanol at 4 °C overnight. Next, 500 μL of propidium iodide (PI)/RNase A was added and incubated with cells for 30 min. Cells were analyzed using a NovoCyte flow cytometer (Agilent, US).

CAOV-3 and OVCAR-3 cells were harvested by trypsinization and incubated in 500 μL binding buffer additionally adding 5 μL AnnexinV-FITC solution and 5 μL PropidiumIodide (PI). Cells were stained in darkness for 15 min and then assessed by flow cytometry. The gating strategy for flow cytometry analysis was shown in the supplementary Fig 3.

### Migration assay

Cell migration assays were performed using 8-μm transwell filters in 24-well plates (LABSELECT, China). In total 5 × 10^3^ CAOV-3 or OVCAR-3 cells in 200 μL of serum-free medium were plated in the upper chamber. A total of 800 μL medium was subsequently added to the lower chamber. The cells were subsequently allowed to incubate for 24 h, and the cells that had migrated to the lower compartment were fixed with 4% paraformaldehyde (Aladdin, Chian) for 20 min and stained with 0.5% crystal violet (Amresco, US) for 5 min. The cells were photographed at 200× magnification, after which the cells in five randomly selected fields in each well were counted.

### Xenograft models in vivo

Experimental animal procedures were approved by the Medical Ethics Committee of Shengjing Hospital Affiliated with China Medical University. Female BALB/c-nu mice (4–6 weeks old) were purchased from Cavens Laboratory Animal Company (China). OVCAR-3 cells (5 × 10^6^ cells) with pLVX-TetOne-Puro-SQLE or Tet-pLKO-Puro-shSQLE system or the appropriate controls were injected into each mouse subcutaneously. The volumes of each xenograft tumor were calculated every three days. Until xenograft tumors were about 150 mm^3^, 1 mg/mL Dox was added to the drinking water of the partial group. After 3 weeks, the mice were euthanized using excessive CO_2_, and tumors were harvested, weighed, and photographed.

### Flow cytometry analysis of cancer stem cell markers in xenograft tumors

The numbers of ALDH^+^CD133^+^ cells and ALDH^-^CD133^-^ cells in the xenograft tumors were detected. Briefly, the single-cell suspensions were prepared from the adherent parental cells and grinding xenograft tumor tissue after filtration. More importantly, the erythrocytes were removed using the Red Blood Cell Lysis Buffer (Solarbio, China). After cleaning with phosphate-buffered saline (PBS) and centrifugation, 1 × 10^6^ cells were resuspended with 100 μL buffer solution, cultured with 0.2 ug ALDH Polyclonal antibody (Proteintech Group, US), following label with CD133 antibody (1:1000) (SANTA, US) for 30 min in darkness. Then the cells were cultured with Cy3-labeled Goat Anti-Mouse IgG antibody (1:50) (Proteintech Group, US) and FITC-labeled Goat Anti-Rabbit IgG antibody (1:1000) (Proteintech Group, US) for 30 min in darkness. The labeled cells were centrifuged and dispersed in 500 μL fluorescence-activated cell sorting (FACS) buffer, which was injected in flow cytometry (Agilent, US) and results were expressed as the percentage of ALDH^+^CD133^+^ cells and ALDH^-^CD133^-^ cells.

### Immunofluorescence (IF) staining

IF staining assays were used to detect the Brdu-label cells in vitro, the level of SQLE and Ki67 in xenograft tumor. After being fixed with 4% paraformaldehyde and permeabilized with 0.1% triton X-100, cells or sections were incubated with BrdU (1:200; Proteintech, China), SQLE (1:100; Abclonal, China), or Ki67 (1:100; Affinity, China) antibody. Then, the cells or sections were incubated with appropriate Cy3-labeled Goat Anti-Rabbit IgG (1:1000; Abcam, US) or Cy3-labeled Goat Anti-Mouse IgG secondary antibody (1:1000; Abcam, US) for another 1 h. Finally, nuclei were stained with DAPI (Aladdin, China), and images were collected by a fluorescence microscope (OLYMPUS, Japan).

### Non-targeted metabolomics analysis by ultrahigh-performance liquid chromatography-electrospray ionization tandem mass spectrometry (UPLC/MS-MS)

A UPLC-MS-based platform from Thermo Fisher Scientific was used to analyze metabolites from OVCAR-3 cells with SQLE overexpression or not. Briefly, cell samples were ground by glass bead and mixed with organic solvent to extract metabolite. The aqueous supernatant was vacuum dried and reconstituted into 300 μL 2-chloro-L-phenylalanine solution. After filtration, 2 μL solution was injected into the UPLC-QTOF-MS system. Chromatographic separation was carried out at 40 °C on an Acquity UPLC HSS T3 column (2.1 mm × 100 mm, 1.8 μm) with a binary gradient at a flow rate of 0.3 mL/min. The mobile phases consisted of 0.1% formic acid in water (solvent A) and 0.1% formic acid in acetonitrile (solvent B) in LC-ESI ( + )-MS analysis, and 5 mM Ammonium formate in water (solvent A) and acetonitrile (solvent B) in LC-ESI (-)-MS analysis. The steps of separation were as follows: 8% B from 0-1 min, 8–98% B from 1–8 min, 98% B from 8–10 min, 98–8% B from 10–10.1 min, 8% B from 10.1–12 min.

The mass spectrometer was operated in both the positive and negative ion modes to detect more metabolites based on the charge bias of metabolites and is suitable for the detection of bodily fluids, cells, and fresh and fixed tissue [50]. Data were acquired in the 100–1000 m/z mass range. Total ion chromatograms were acquired according to the following operation parameters: Capillary voltages of +3.5 kV and −2.5 kV for the positive and negative modes, a source temperature of 325 °C. Data from the MS/MS analyses were acquired by Proteowizard (v3.0.8789) package and multiple metabolic databases.

Principal component analysis (PCA) and orthogonal partial least squares discriminant analysis (OPLS-DA) were performed to assess the discriminating power of metabolite datasets in unsupervised and supervised manners, respectively. Following log transformation and variance normalization of the metabolite levels, intergroup fold differences (FC) for SQLE overexpression samples vs control samples were generated and expression differed significantly between compared groups were analyzed. The differentially expressed metabolites (DEMs) were selected according to the value of FC, p, and variable importance in projection (VIP).

### Methylated RNA immunoprecipitation-qPCR (MeRIP-qPCR) assays

Total RNA was extracted from cells by TRIpure lysis buffer, and then treated with DNase (Sigma, US) to remove genomic DNA. The mRNA fragments were incubated with m^6^A primary antibody for immunoprecipitation using a riboMeRIPTM m^6^A transcriptome profiling kit (Ribobio, China). Both input control and m^6^A-IP samples were subjected to qRT-PCR with gene-specific primers. The primers for MeRIP-qPCR are listed in supplementary Table 4.

### mRNA stability assay

CAOV-3 and OVCAR-3 cells with WTAP overexpression or FTO knockdown were collected. After that, 5 mg/mL actinomycin D (MedChemExpress, US) was used to treat cells for 0, 1, 2, 3, and 4 h. Then, cells were lysed and the total RNA was extracted and analyzed using qRT-PCR.

### RNA-binding protein immunoprecipitation (RIP)

RIP assays were performed using a Magna RIP^TM^ RNA-Binding Protein Immunoprecipitation Kit (Millipore) according to the manufacturer’s protocol. Briefly, Antibodies of SQLE (5 μg) were pre-bound to protein A/G magnetic beads in immunoprecipitation buffer for 2 h and then incubated with 100 μL of cell lysate. RNA was eluted from the beads by incubation and dissolved in RNase-free water. Finally, collected RNAs were transformed into cDNA and then detected by agarose gel electrophoresis.

### Statistical analysis

Data are expressed as means with corresponding standard deviations (SD). A minimum of three independent experiments were performed with similar results. GraphPad Prism software was used for statistical analyses. Correlations between gene expression and clinicopathological data were analyzed using χ2 and Fisher’s exact tests, respectively. Survival curves were generated using the Kaplan-Meier method. Differences between two groups were analyzed using two-tailed unpaired Student’s t-tests between two groups, and differences in three or more three groups were analyzed using one-way ANOVA followed by Bonferroni test for multiple comparisons. A *p*-value < 0.05 was set as the threshold for statistical significance. *p < 0.05, **p < 0.01. and ***p < 0.001.

## Supplementary information

Final Supplementary Materials

Original full length blots

## Data Availability

The dataset(s) supporting the conclusions of this article is(are) included within the article (and its additional file(s)).
