# Supplementary material for: Stabilization of SQLE mRNA by WTAP/FTO/IGF2BP3-dependent manner in HGSOC: implications for metabolism, stemness, and progression
Source: Cell Death Dis. 2024 Dec 1;15(12):872. doi: 10.1038/s41419-024-07257-6 (PMC11609299; doi:10.1038/s41419-024-07257-6)
Supplement: Supplementary file 1 — Final Supplementary Materials [file 41419_2024_7257_MOESM1_ESM.docx]

Supplementary Table 1 The primers used in the qRT-PCR analysis were shown.

| Genes | forward primer | reverse primer |
| --- | --- | --- |
| SQLE | AACGAGGTGTTCTTCTTTTG | CAGGGAATCATCTGTGGC |
| ALDH | GGCAGCCATTTCTTCTCA | CTTCTTAGCCCGCTCAAC |
| CD133 | CCAAGGACAAGGCGTTCA | GCACCAAGCACAGAGGG |
| WTAP | ACTAAAGCAACAACAGCAGG | CGTAAACTTCCAGGCACTC |
| FTO | GAACACCAGGCTCTTTACG | ATGAACCCATCCCAACC |
| IGF2BP1 | CCATCCGCAACATCACAA | TTCAGGGGAACCTCGTCA |
| IGF2BP2 | TGAAACAGGGACCAAGATA | GTGGAGATAGCACGGACA |
| IGF2BP3 | AGGGAGTCTTATGAAAATGAT | ATGGCACCGACTGATAGA |
| GAPDH | GACCTGACCTGCCGTCTAG | AGGAGTGGGTGTCGCTGT |

Supplementary Table 2 The antibodies used in the WB assay were shown.

| Primary antibodies | Dilution rate | Incubation condition | manufacturers |
| --- | --- | --- | --- |
| Anti-SQLE | 1:1000 | 4℃ overnight | ABclonal, Wuhan, China |
| Anti-WTAP | 1:1000 | 4℃ overnight | ABclonal, Wuhan, China |
| Anti-FTO | 1:1000 | 4℃ overnight | ABclonal, Wuhan, China |
| Second antibodies |  |  |  |
| Goat Anti-Rabbit IgG/HRP | 1:3000 | 37℃ 1 h | Solarbio, Beijing, China |
| Goat Anti-Mouse IgG/HRP | 1:3000 | 37℃ 1 h | Solarbio, Beijing, China |

Supplementary Table 3 The target sequence of shRNA or siRNA used in this study

| Name | Sequence (Positive chain:5´-3´) |
| --- | --- |
| shSQLE | tgctgttgacagtgagcgcgcctgttttctttatttcaaatagtgaagccacagatgtatttgaaataaagaaaacaggcttgcctactgcctcgga |
| siWTAP | GCGAAGUGUCGAAUGCUUATT |
| siFTO | GGAAGAAGAUGGAGGGUGUTT |
| siIGF2BP1 | GGCCCAUAAUAACUUUGUATT |
| siIGF2BP2 | UGAAGCUGGAAGCGCAUAUTT |
| siIGF2BP3 | GUGAAUGAACUUCAGAAUUTT |

Supplementary Table 4 The primers for SQLE used in the MeRIP-qPCR analysis were shown.

| region | forward primer | reverse primer |
| --- | --- | --- |
| 3’UTR | AGGGGAACCATTTGTGA | TCTAACTTATTTAACCCCTTCA |
| CDS | GTGGACTTTTCTGGGCATT | AGAGGGCGAACTGGGAG |


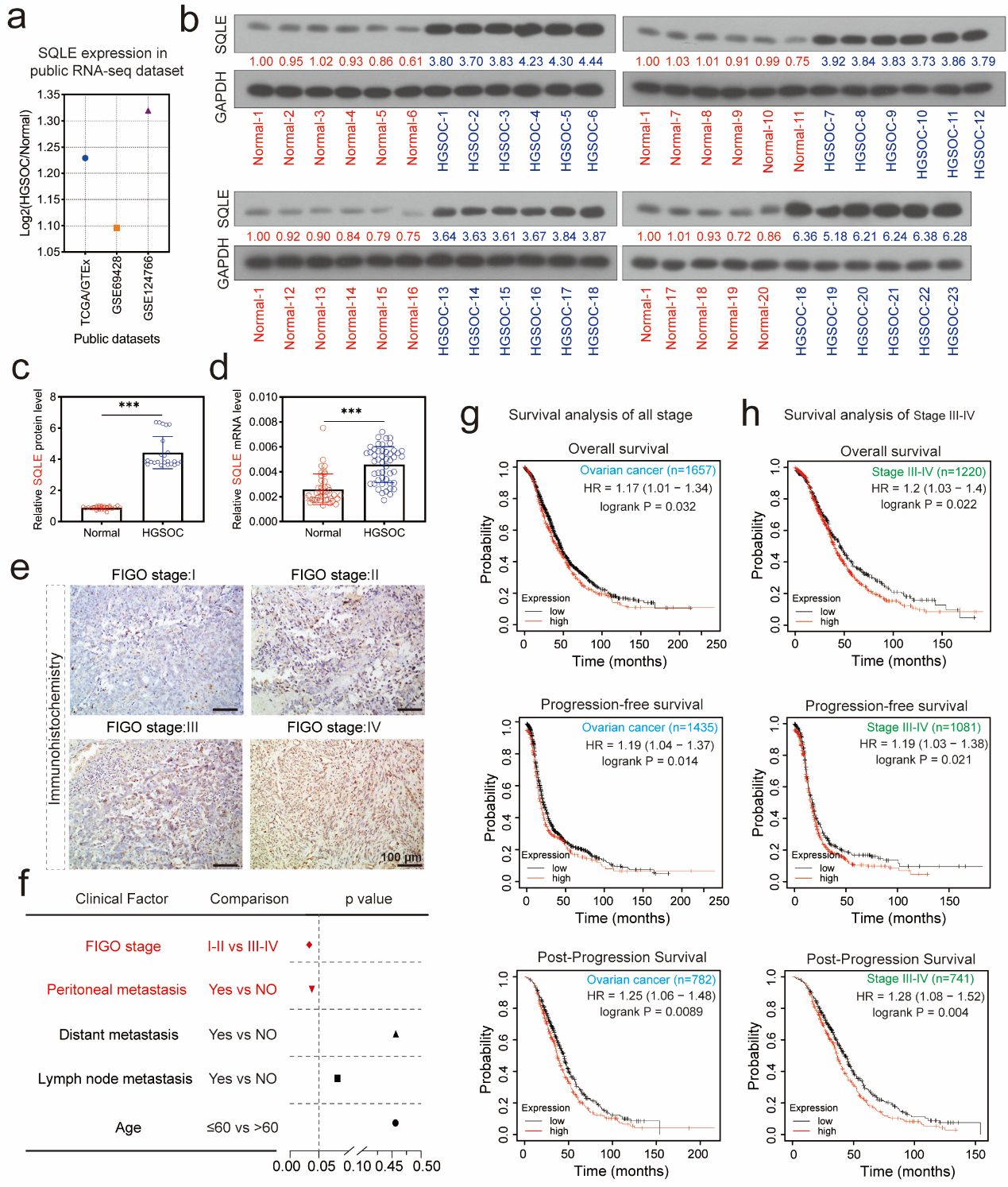


**Supplementary Figure 1 SQLE was highly expressed in clinical HGSOC samples and predicted poor prognosis of HGSOC patients.** (a) The SQLE expression in public datasets, such as TCGA/GTEx (489 ovarian serous adenocarcinoma samples from TCGA and 88 noramal ovarian samples from GTEx dataset), GSE69428 (10 HGSOC samples and 10 normal ovarian samples) and GSE124766 (8 HGSOC samples and 3 normal ovarian samples). (b-c) WB assays verified the high level of SQLE in part clinical samples. (d) The qRT-PCR assays were conducted to detect the level of SQLE in normal samples and HGSOC samples. (e) Immunohistochemistry analysis of HGSOC samples in FIGO stage (Ⅰ-Ⅳ). Original magnifications for immunohistochemistry were 200×. (f) The chi-square test analyzed the relationship between SQLE expression and clinicopathological features of HGSOC. (g-h) Kaplan-Meier survival analysis about overall survival (OS), progression-free survival (PFS), and post-progression survival (PPS) (<http://kmplot.com/analysis/index.php?p=background>) that upregulation of SQLE level was related to poor prognosis of ovarian cancer samples and HGSOC samples (HR > 1, logrank P < 0.05). **p* < 0.05, ***p* < 0.01, and ****p* < 0.001.


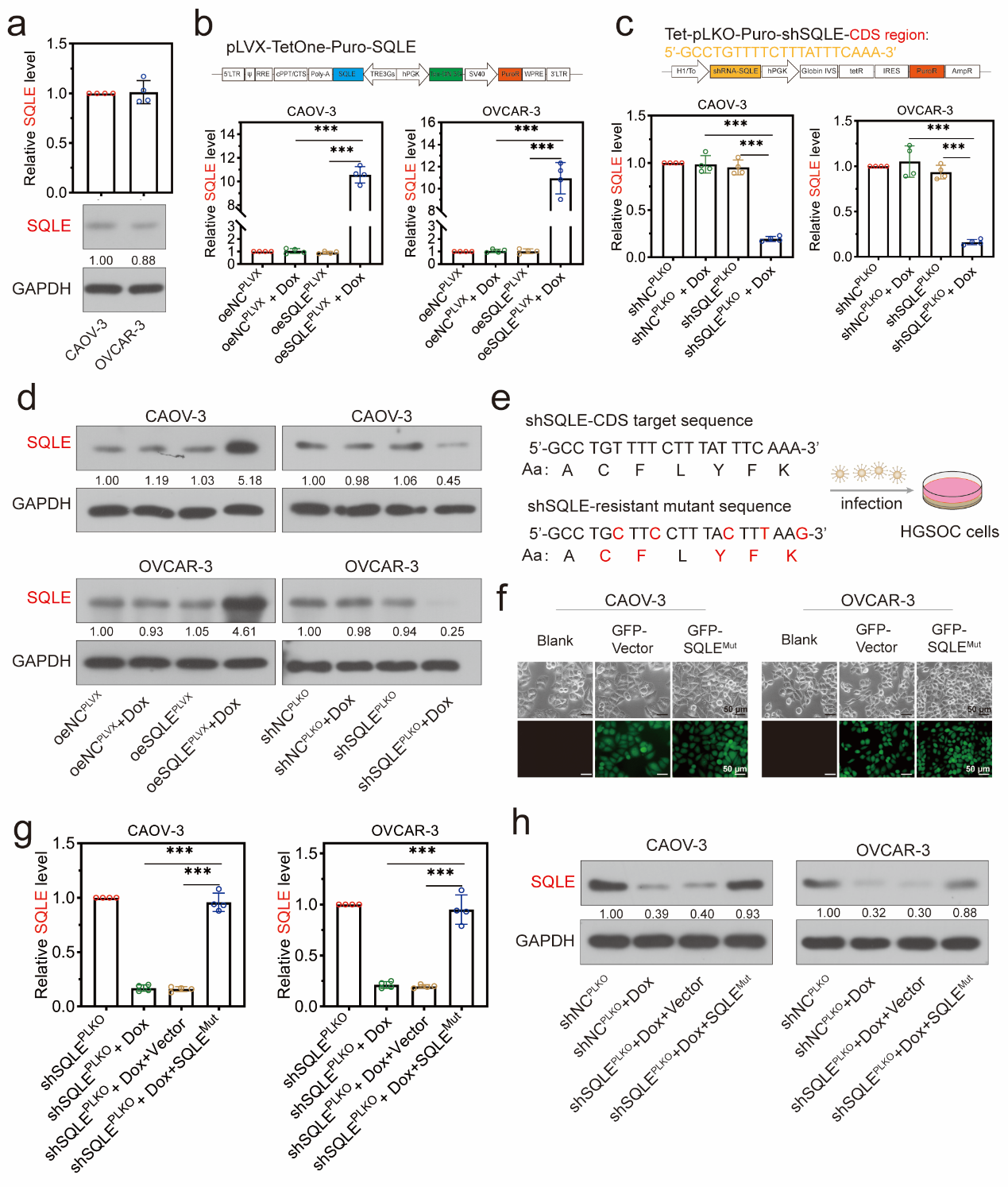


**Supplementary Figure 2 The dox-inducible lentiviral vectors specifically induced the SQLE overexpression and knockdown in HGSOC cells.** (a) There was no significant difference in intracellular SQLE expression from CAOV-3 and OVCAR-3 cells. (b) The structure of pLVX-TetOne-Puro-SQLE vectors carrying the CDS region of SQLE was shown. Dox treatment activated the vectors and induced SQLE overexpression in CAOV-3 and OVCAR-3 cells. (c) The structure of Tet-pLKO-Puro-shSQLE vectors targeting the CDS region of SQLE was shown. Dox treatment activated the vectors and induced SQLE knockdown in CAOV-3 and OVCAR-3 cells. (d) The expression of SQLE protein in lentivirus-infected cells was verified by WB assays. (e) Schematic representation of the GFP-labeled expression vectors carrying the silent mutation of SQLE. The shSQLE target sequence (encoding ACFLYFK amino acids) is indicated by capital letters. Mutations introduced are indicated by red letters. (f) GFP fluorescence was observed in CAOV-3 and OVCAR-3 cells transfected with GFP-SQLE-mutation expression constructs. Original magnifications were 200×. (g-h) The SQLE mRNA and protein levels from CAOV-3 and OVCAR-3 cells with shSQLE or/and SQLE-mutation expression were detected by qRT-PCR analysis and WB assays. The SQLE expression was recovered in the rescue experiment using GFP-SQLE mutation. **p* < 0.05, ***p* < 0.01, and ****p* < 0.001.


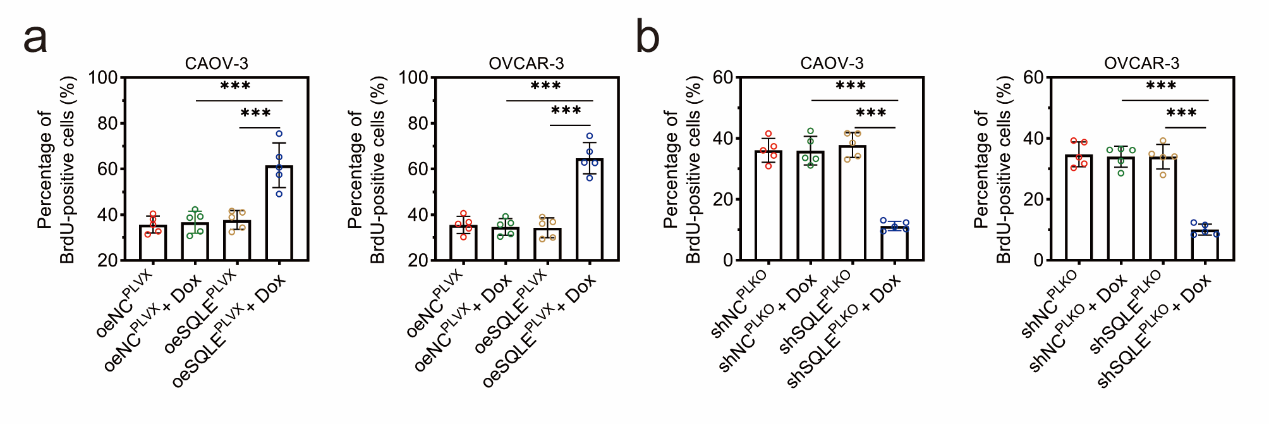


**Supplementary Figure 3 The SQLE promotes HGSOC cell proliferation *in vitro*.** (a) SQLE overexpression significantly increased the percentage of BrdU-positive cells. (b) SQLE knockdown significantly decreased the percentage of BrdU-positive cells .


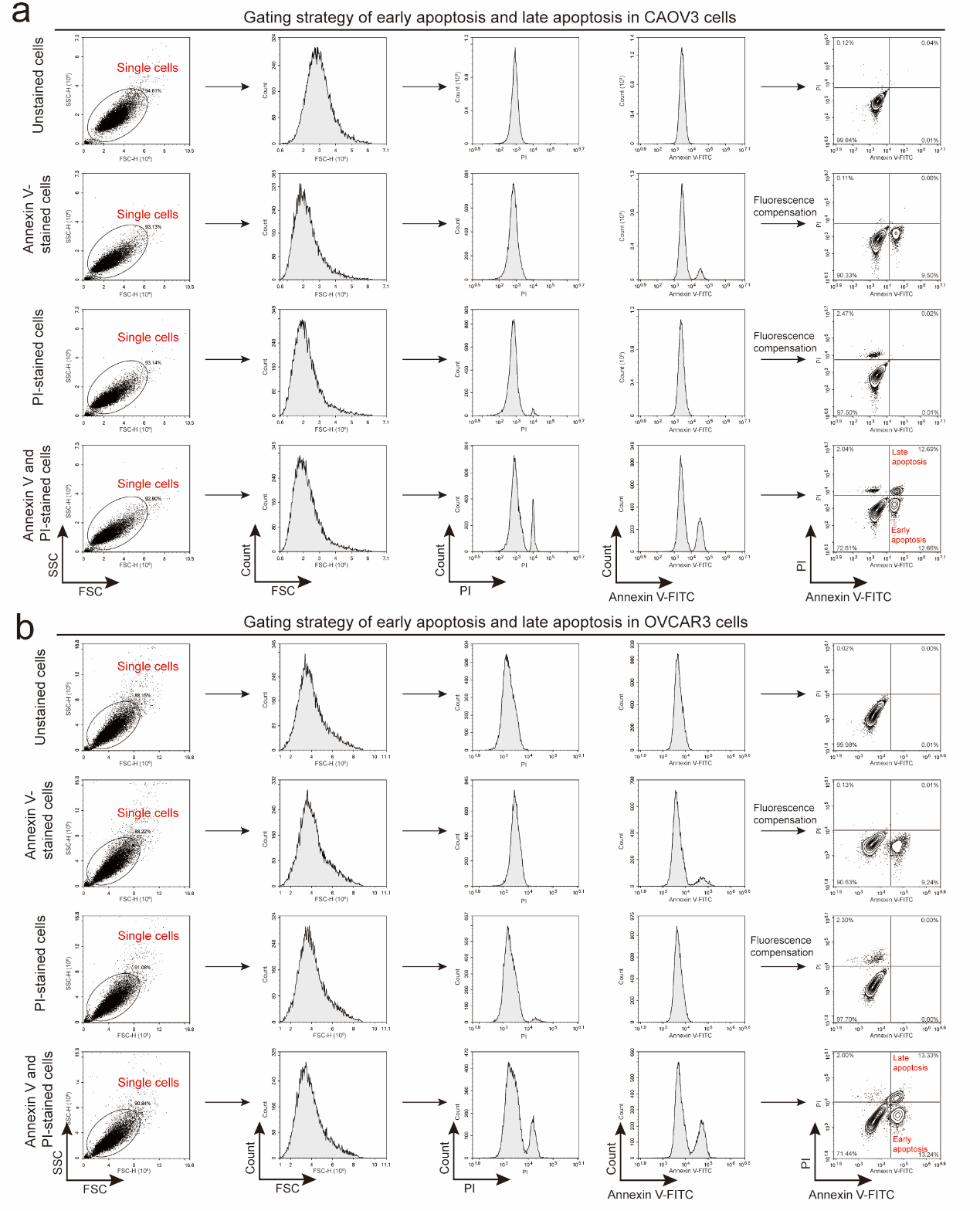


**Supplementary Figure 4 Gating strategy for flow cytometry analysis of early apoptosis and late apoptosis in CAOV-3 and OVCAR-3 cells.** (a-b) The unstained cells, cells that were only stained with Annexin V-FITC, and cells that were only stained with PI serve as controls. Before loading the experimental samples, control groups were used to adjust voltage and fluorescence compensation. For experimental samples with double staining of Annexin V and PI, the single-cell population in bivariate scatter plots was identified by gating FSC/SSC. The histogram further shows that the single cell population as the main cell population appears as a large peak. Annexin V-positive gate and PI-positive gate were used to determine according to cell population distribution in the bivariate contour plots. The corresponding histograms were used to judge the accuracy. The fluorescence intensity of Annexin V cross-arranged with the fluorescence intensity of Annexin V, divided the site into four quadrants. Annexin V-positive and PI-positive cells were seen as late apoptosis, and Annexin V-positive and PI-negative cells were seen as early apoptosis, respectively.


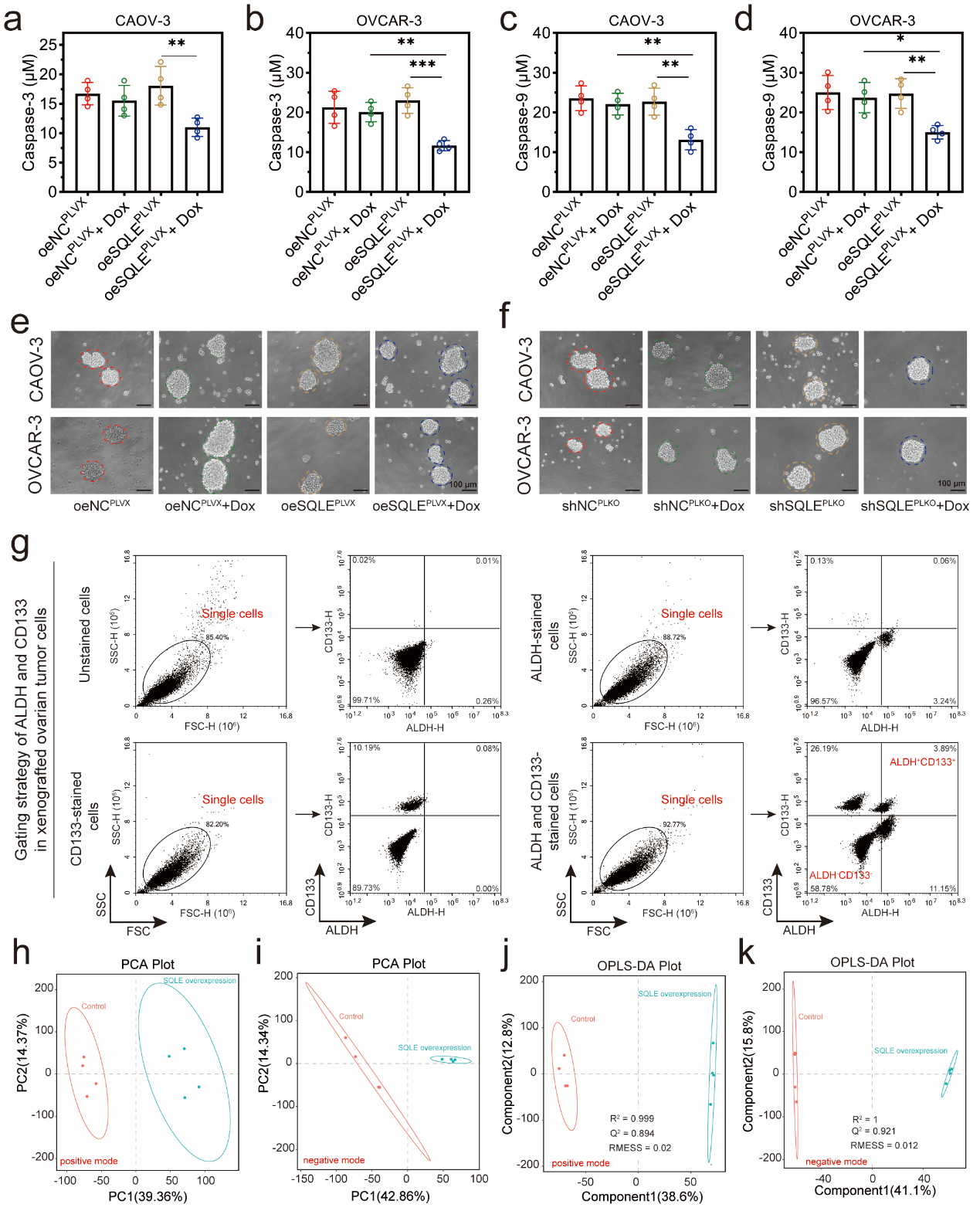


**Supplementary Figure 5 SQLE-related apoptosis, stemness, and metabonomics markers.** (a-b) The levels of caspase-3 in CAOV-3 and OVCAR-3 cells with SQLE overexpression or not were detected. (c-d) The levels of caspase-9 in CAOV-3 and OVCAR-3 cells with SQLE overexpression or not were detected.(e-f) The images of CAOV-3 or OVCAR-3-derived tumorspheres were captured by inverted microscopy at a magnification of × 100. (g) Cancer stem cell markers in xenograft-derived ovarian cancer cell lines. Xenograft-derived tumor cells were stained for stem cell markers. Results were expressed as the percent of ALDH^+^CD133^+^ or ALDH^-^CD133^-^ cells in total tumor cells. (h-i) The non-targeted metabolomic data was carried out by principal component analysis (PCA) in positive mode and negative mode. (j-k) The orthogonal projections to latent structures-discriminant analysis (OPLS-DA) were conducted to examine differences between HGSOC samples and normal ovarian tissue. PLS-DA score plots in positive mode and negative mode were shown. The root mean square error of estimation (RMESS) was low, suggesting that the model's sensitivity, accuracy, and specificity were all 100%. **p* < 0.05, ***p* < 0.01, and ****p* < 0.001.


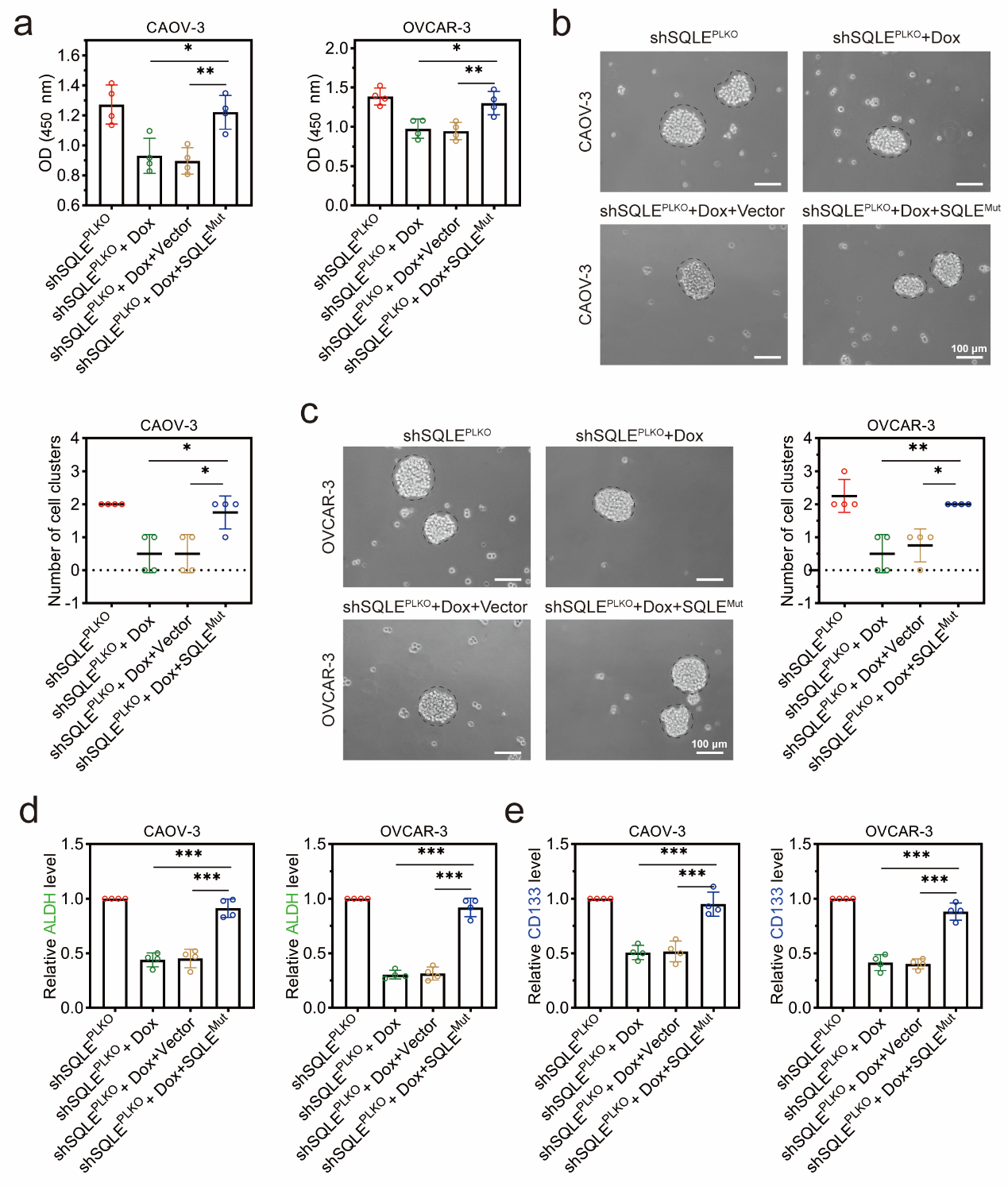


**Supplementary Figure 6 SQLE knockdown phenotype was rescued by expression of shRNA-resistant mutant SQLE.** (a) SQLE knockdown induced the decrease of CAOV-3 and OVCAR-3 cells, which was reversed after transfecting with GFP-SQLE-mutation expression constructs. (b-c) The shRNA-resistant GFP-SQLE rescued the stemness failure phenotype in cells transfected with shSQLE. The typical pictures of tumorspheres were shown. The magnification of the pictures was 100×. (d-e) The qRT-PCR analysis of ALDH expression and CD133 expression in tumorspheres. **p* < 0.05, ***p* < 0.01, and ****p* < 0.001.


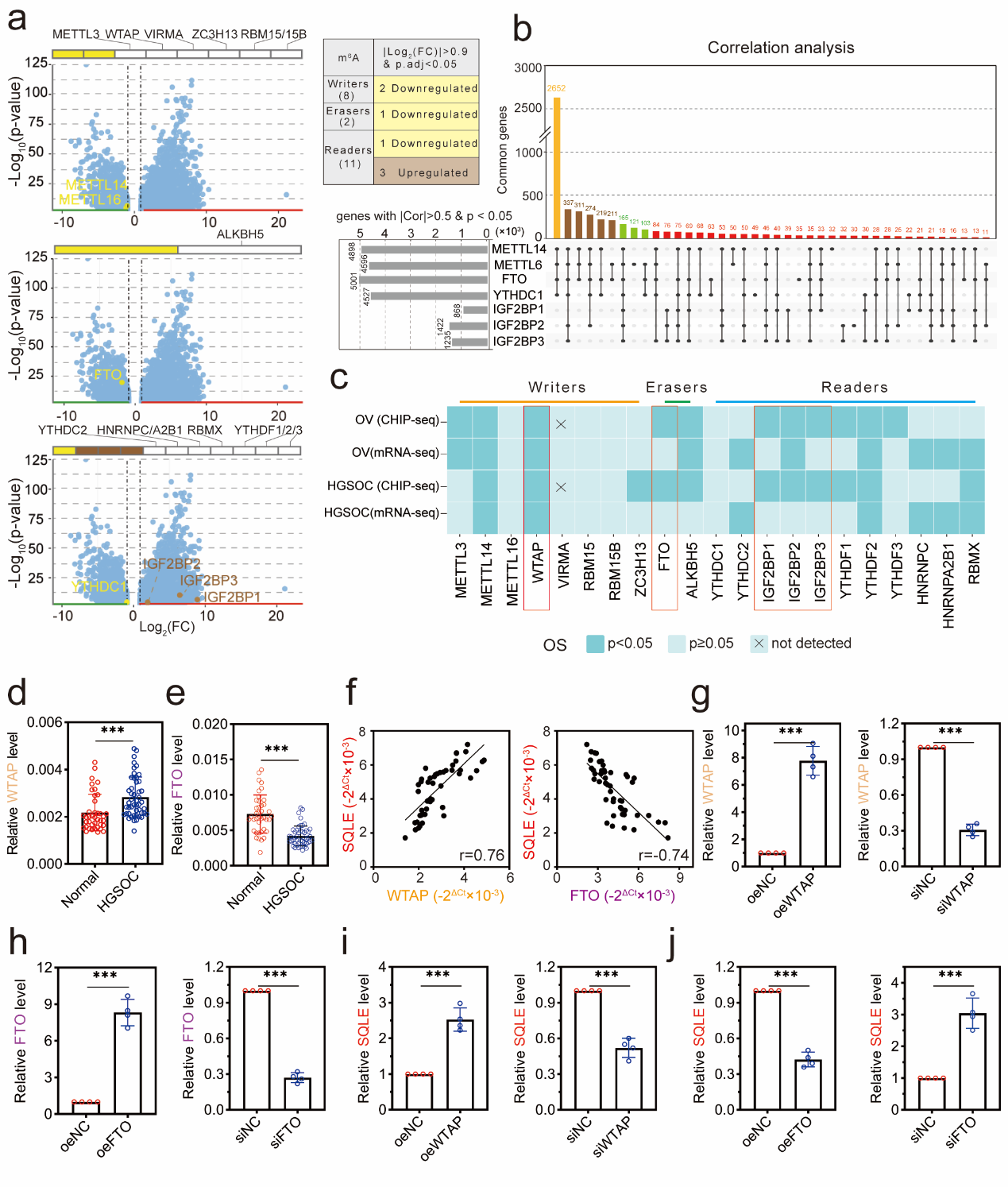


**Supplementary Figure 7 The SQLE level was related to m^6^A regulators.** (a) The volcano maps showed the expression of “Writers”, “Erasers”, and “Reader” respectively. (b) The correlation analysis between differentially expressed m^6^A regulator and DEGs (|Log_2_FC|>1 & p<0.05). (c) The analysis of overall survival for patients with HGSOC was carried with the KMplot website based on the public ChIP sequencing data and mRA sequencing data. Expression levels of the m^6^A regulator were associated with OS (p < 0.05) in covariate-adjusted single-gene analyses. (d-e) The expression of WTAP and FTO in clinical samples with HGSOC and normal ovary. (f) The correlation analysis between SQLE level and WTAP level, and between SQLE level and FTO level were calculated. (g-h) The WTAP and FTO were efficiently overexpressed and silenced. (i) The SQLE level was positively regulated by the WTAP level. (j) The SQLE level was negatively regulated by the FTO level. **p* < 0.05, ***p* < 0.01, and ****p* < 0.001.


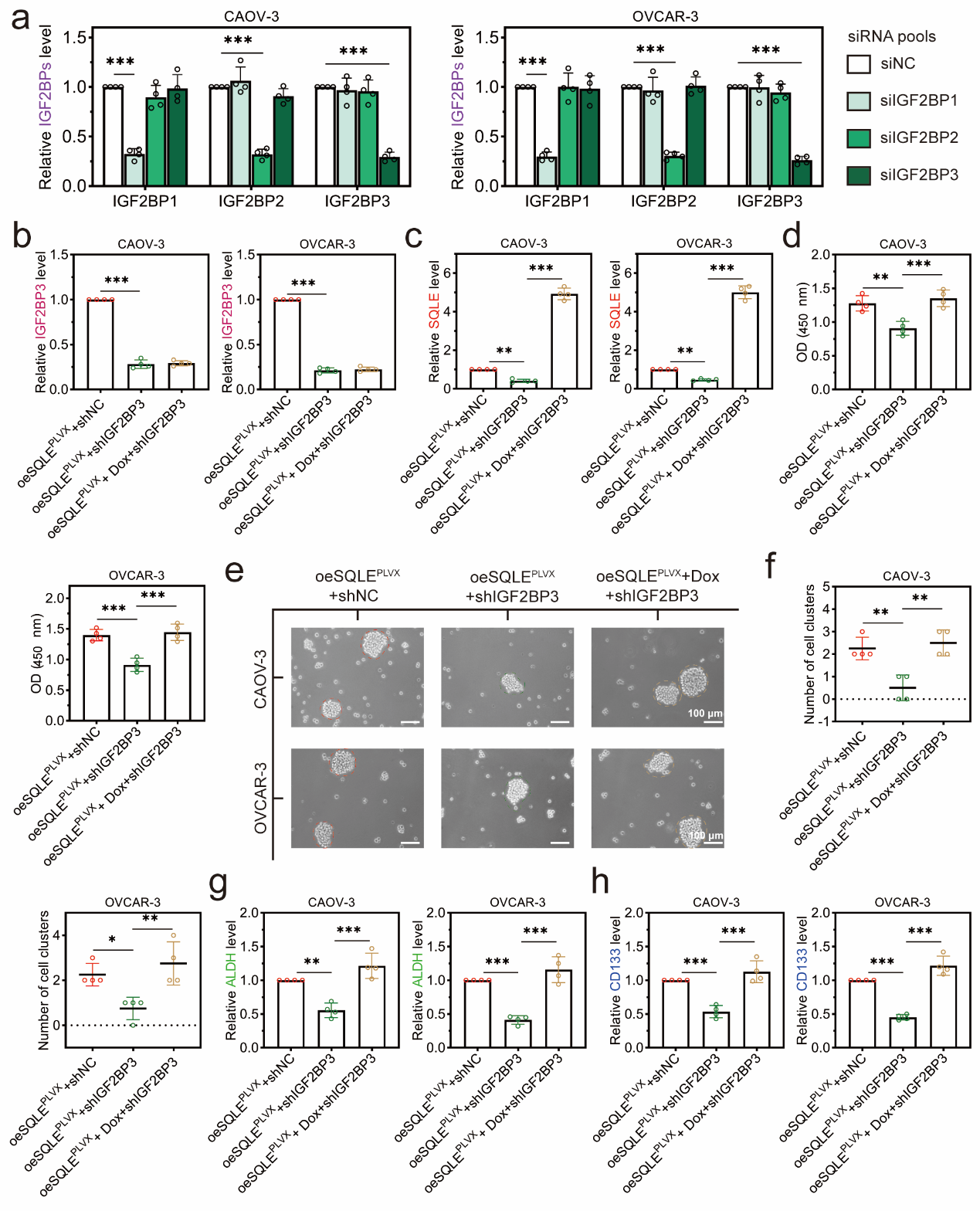


**Supplementary Figure 8 IGF2BP3 contributes to the functional phenotype of the cell induced by SQLE.** (a) The IGF2BP1/2/3 were specifically silenced in CAOV-3 and OVCAR-3 cells. (b) The knockdown of IGF2BP3 in CAOV-3 and OVCAR-3 cells with SQLE overexpression or not. (c) IGF2BP3 knockdown induced the decrease of SQLE expression, and Dox treatment recovered the SQLE expression by activating the inducible SQLE expression vectors. (d) The ability of cells with IGF2BP3 knockdown and cells with IGF2BP3 knockdown and SQLE overexpression was detected. (e-f) The sphere formation assays confirmed that the inhibition of sphere formation ability induced by IGF2BP3 knockdown was recovered by SQLE overexpression. (g-h) The ALDH and CD133 mRNA levels in tumorspheres were detected by the qRT-PCR analysis. **p* < 0.05, ***p* < 0.01, and ****p* < 0.001.
