# Supplementary material for: Stabilization of SQLE mRNA by WTAP/FTO/IGF2BP3-dependent manner in HGSOC: implications for metabolism, stemness, and progression
Source: Cell Death Dis. 2024 Dec 1;15(12):872. doi: 10.1038/s41419-024-07257-6 (PMC11609299; doi:10.1038/s41419-024-07257-6)

Figure 4c

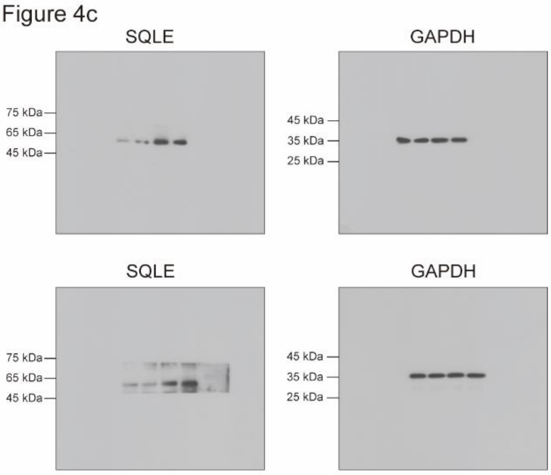

Figure 4g

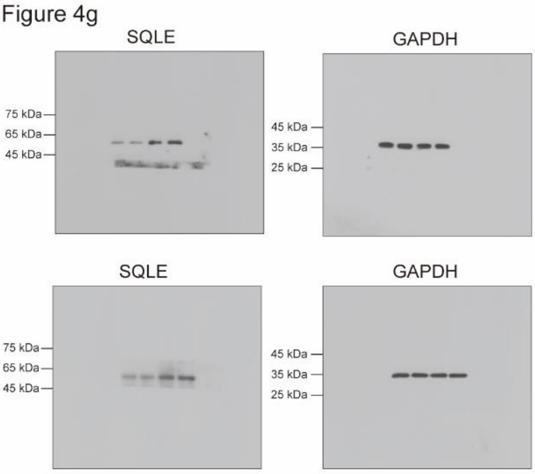

Figure 7c

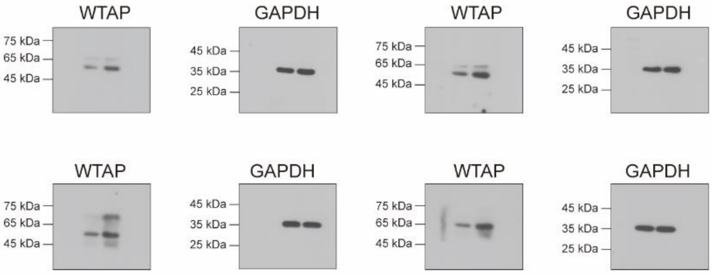

Figure 7f

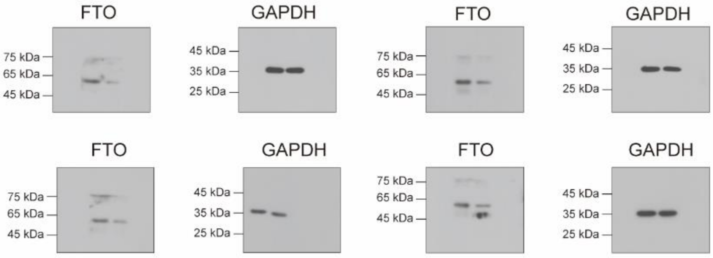

Figure 8e

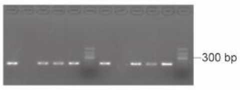

Figure 8i

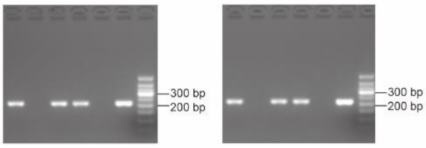

Figure 8m

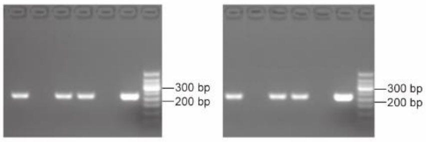

Supplementary materials Figure 1b

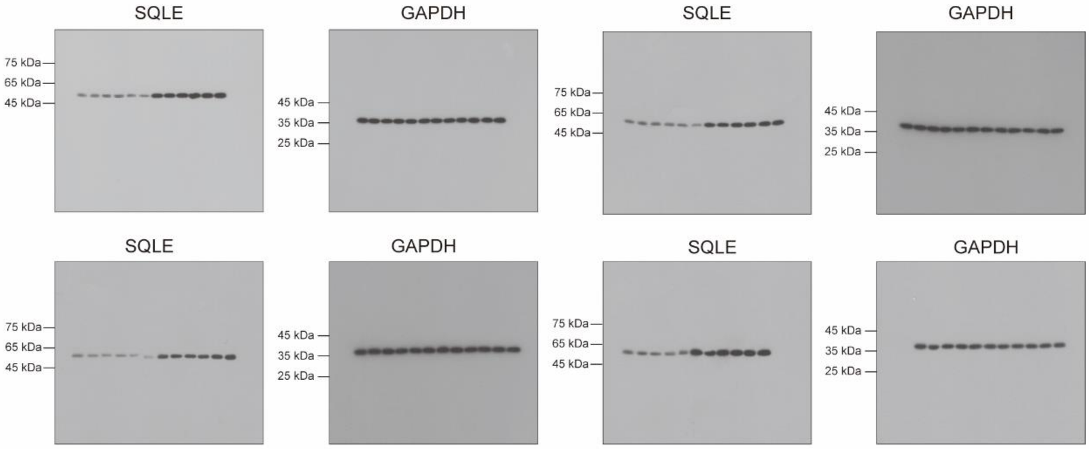

Supplementary materials Figure 2a

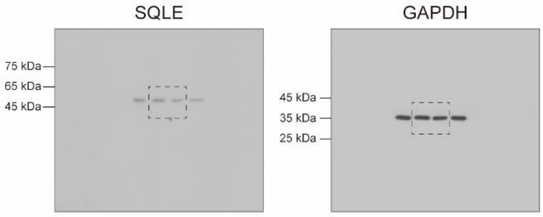

Only the second and third lane (Inside the dotted box) were shown in the Supplementary materials Figure 2a

Supplementary materials Figure 2d

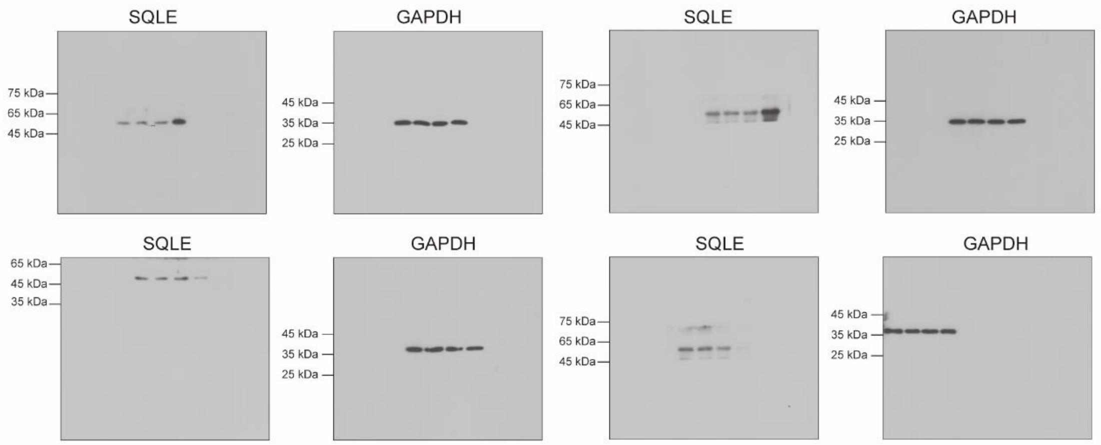

Supplementary materials Figure 2h

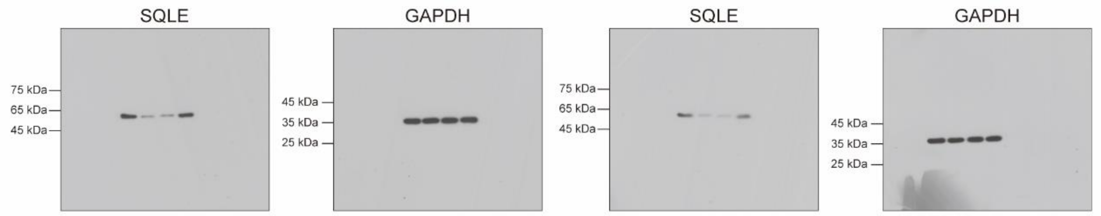

Supplement: Supplementary file 2 — Original full length blots [file 41419_2024_7257_MOESM2_ESM.pdf]
